# Microbial-caddisfly bioherm association from the Lower Cretaceous Shinekhudag Formation, Mongolia: Earliest record of plant armoring in fossil caddisfly cases

**DOI:** 10.1371/journal.pone.0188194

**Published:** 2017-11-21

**Authors:** Tsolmon Adiya, Cari L. Johnson, Mark A. Loewen, Kathleen A. Ritterbush, Kurt N. Constenius, Cory M. Dinter

**Affiliations:** 1 Department of Geology and Geophysics, University of Utah, Salt Lake City, Utah, United States of America; 2 Natural History Museum of Utah, Salt Lake City, Utah, United States of America; 3 Evolutionary Studies Institute, University of the Witwatersrand, Johannesburg, South Africa; 4 Department of Geosciences, University of Arizona, Tucson, Arizona, United States of America; 5 School of Geosciences, University of Witwatersrand, Johannesburg, South Africa; Indiana University Bloomington, UNITED STATES

## Abstract

Caddisfly larvae construct underwater protective cases using surrounding materials, thus providing information on environmental conditions in both modern and ancient systems. Microbial bioherms associated with caddisfly cases are found in the Berriassian-Hauterivian (~140–130 Ma) Shinekhudag Formation of Mongolia, and yield new insights into aspects of lacustrine paleoecosystems and paleoenvironments. This formation contains the earliest record of plant-armored caddisfly cases and a rare occurrence of microbial-caddisfly association from the Mesozoic. The bioherms are investigated within the context of stratigraphic correlations, depositional environment interpretations, and basin-evolution models of the sedimentary fill. The bioherms form 0.5–2.0 m diameter mound-shaped bodies and are concentrated within a single, oil shale-bound stratigraphic interval. Each bioherm is composed of up to 40% caddisfly cases along with stromatolites of millimeter-scale, micritic laminations. Petrographic analyses reveal these bioherms are composed of non-systematic associations of columnar and oncoidal microbialites, constructed around colonies of caddisfly cases. The cases are straight to curved, slightly tapered, and tube-shaped, with a progressively increasing length and width trend (7–21 mm by 1.5–2.5 mm). Despite these variations, the case architectures reveal similar construction materials; the particles used for cases are dominated by plant fragments, ostracod valves, carbonate rocks, and rare mica and feldspar grains. Allochems within the bioherms include ooids, ostracods, plant fragments, rare gastropods, feldspar grains bound in micritic matrices, and are consolidated by carbonate dominated cements. The combination of microbial-caddisfly association, plant fragment case particles, and ooids/oncoids are indicative of a shallow, littoral lake setting. Stratigraphic juxtaposition of nearshore bioherms and the bounding distal oil-shale facies suggests that the bioherms developed in an underfilled lake basin, resulting from an abrupt and short-lived lake desiccation event. Lake chemistry is believed to have been relatively alkaline, saline to hypersaline, and rich in Ca, Mg, and HCO_3_ ions. Through analyzing bioherm characteristics, caddisfly case architecture, carbonate microfacies, and stratigraphic variability, we infer larger-scale processes that controlled basin development during their formation.

## Introduction

Fossil insects are well-known in geological records and, in addition to their entomological and paleontological implications, have also been used as paleoenvironmental indicators, particularly in lacustrine systems [1,2,3,4,5]. Out of millions of insect taxonomic orders, the caddisflies are distinguished by their adaptive and innovative behavior. Caddisflies are fully aquatic during their larval and pupal stage, when they typically build hard cases to cover their soft bodies, using materials from their immediate surrounding aquatic environment [6]. Fossil caddisflies and their cases appear as early as Triassic time, both in aquatic and terrestrial realms, surviving the major Mesozoic to Cenozoic extinction events and aftermaths [7]. The creative construction of hard cases for protection and respiration purposes is critical to caddisflies’ survival and adaptation skills [8].

Multiple Early Cretaceous examples are documented from Asia including China, Korea, and Mongolia [7,9,10]. However, our current knowledge of caddisflies is mainly limited to Eocene and younger examples (e.g., Eocene Green River Formation, Oligocene Limestone Formation) due to excellent exposures and preservation in these sections [11,12,13].

Case studies of the Eocene Green River Formation in Wyoming, USA have demonstrated the effectiveness of fossil caddisfly case analysis for sedimentary facies predictions and paleoenvironmental reconstructions [14]. Here, thousands of caddisfly cases are found within a thick sequence of microbially-influenced lacustrine carbonates [15,16]. Generally, carbonate facies of the lacustrine systems are altered by both biological and depositional processes, and thus these strata contain important insights to the paleoecosystem and paleoenvironment [17,18]. In the Green River Formation, the co-existence of fossil caddisfly cases and microbialites was used to infer a depositional model for carbonate bioherm facies [13,19,20], because each of these biologic components (caddisfly and microbialite) have environmental preferences and tolerances including light, oxygen, and nutrient availabilities that are fundamentally dependent on wave energy, bathymetry, lake hydrology, and lake chemistry [21,22,23]. Additionally, the debris incorporated in the caddisfly larval cases indicates the main detrital components of the environment [13]. Therefore, paleoenvironment interpretations require detailed evaluation of the caddisfly cases.

**Fig 1:**
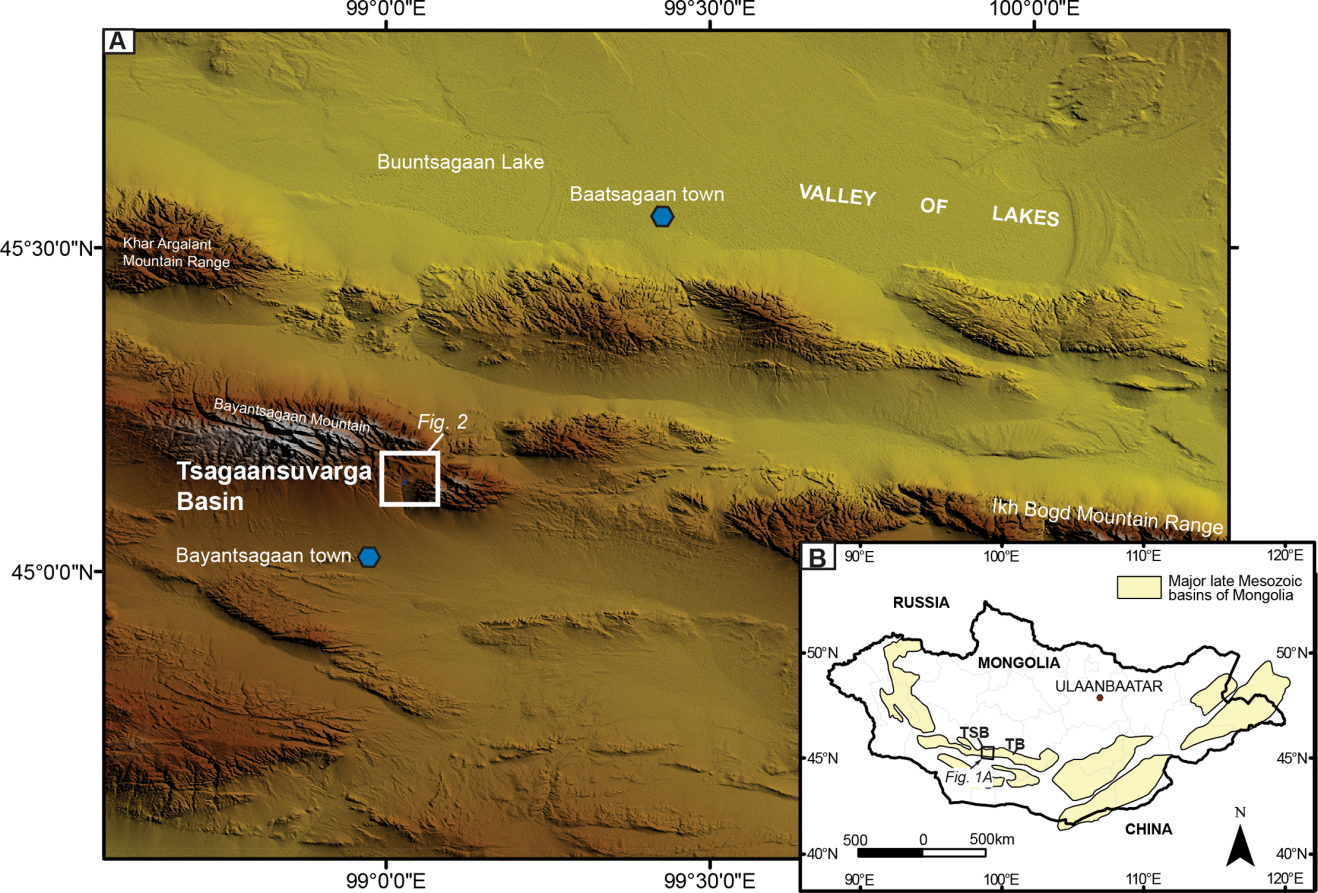
Tsagaansuvarga Basin location and Mesozoic sedimentary basins of Mongolia. (A) Tsagaansuvarga Basin location map overlain on the regional digital elevation model. (B) Mesozoic sedimentary basins of Mongolia.

Here we present a study from the Early Cretaceous Tsagaansuvarga Basin of Mongolia (Fig 1). The carbonate bioherm facies, similar to those in the Green River Formation, are found within lacustrine basin fill and provide an excellent opportunity to test the use of fossil caddisfly case analysis for carbonate facies interpretation and paleoenvironmental reconstructions. Using these interpretations from the basin, we reveal overall evolution of the lake basin in response to larger scale processes. Additionally, we aim to improve the knowledge base of the underwater behavior and evolution of the caddisfly through investigating their case architecture.

### Geological Background of the Tsagaansuvarga area

**Fig 2:**
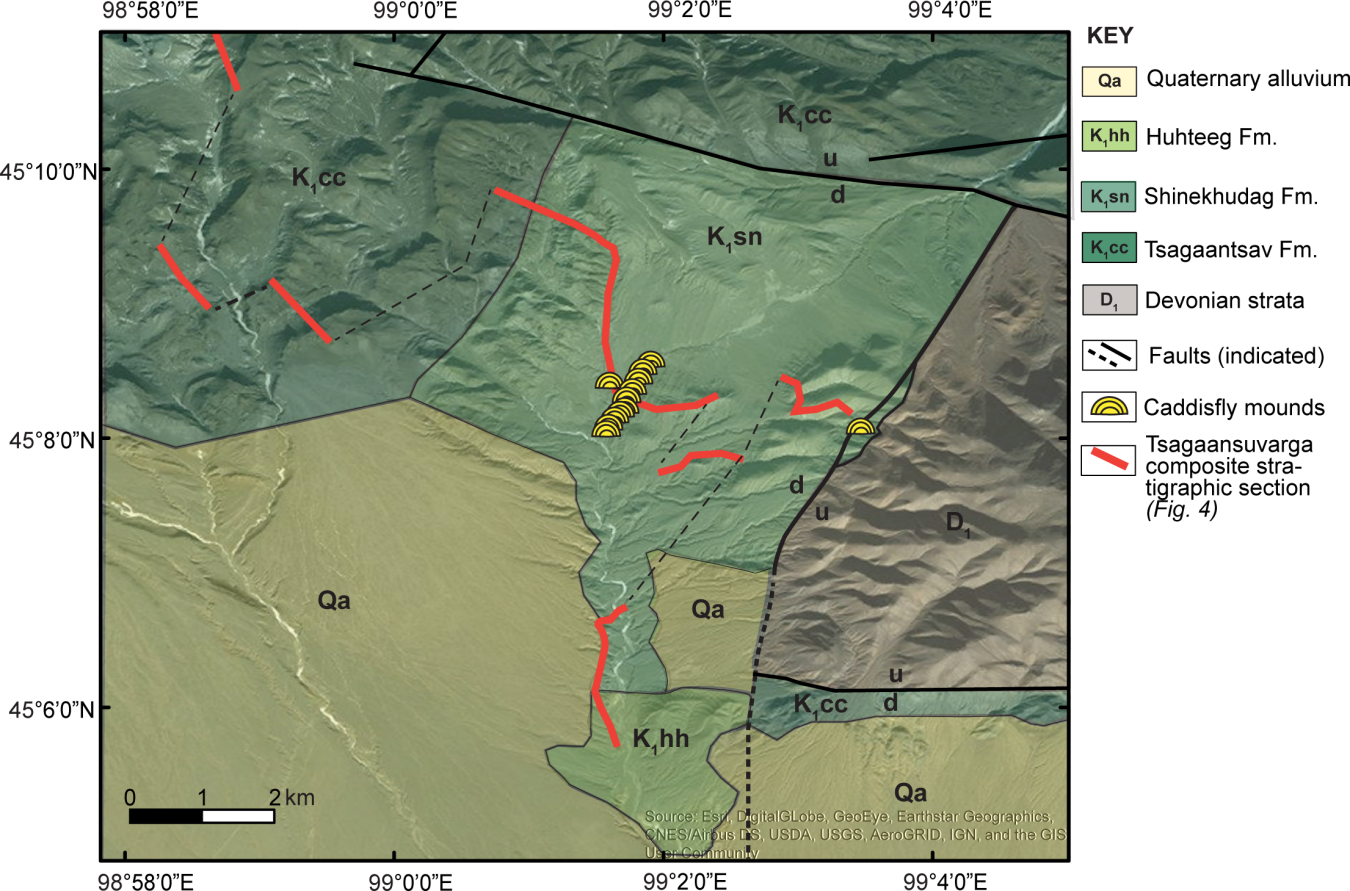
Local geological map overlain on the satellite imagery (modified after Zabotkin et al., 1988). Refer to the keys for map units and symbols. Fm: formation.

**Fig 3:**
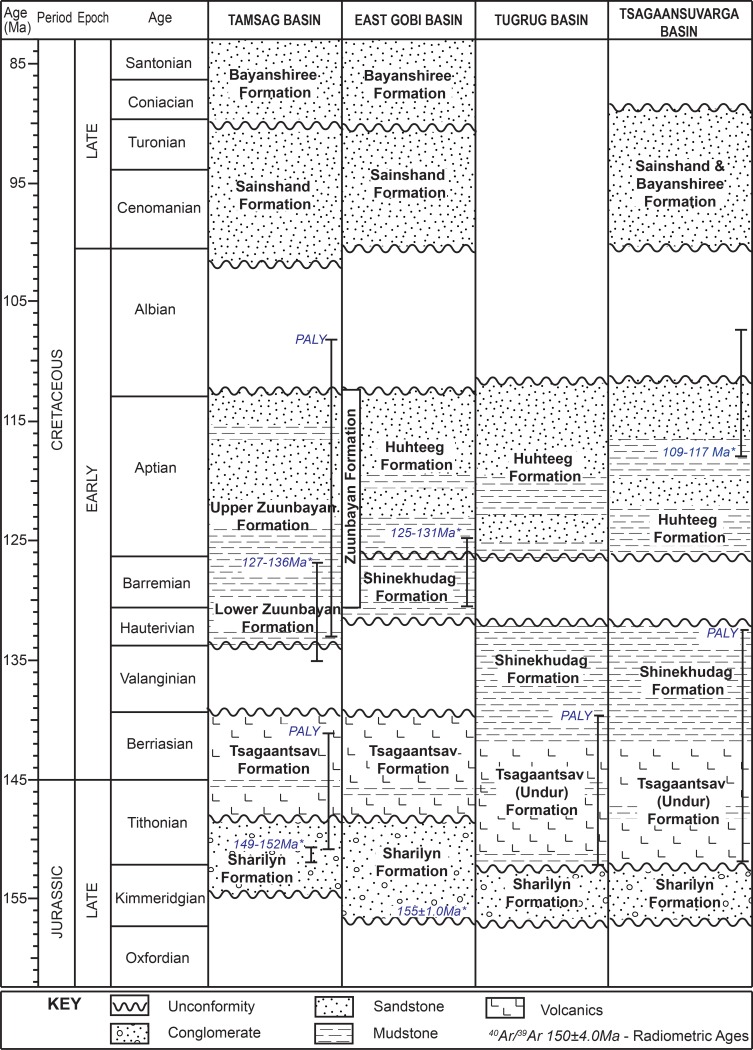
Regional stratigraphic correlation of Late Mesozoic of Mongolia (modified after Barry, 1999; Graham et al., 2001; Johnson et al, 2004; Horton et al., 2013; Petromatad, written communication, 2017). *- ^40^Ar/^39^Ar absolute ages; PALY- palynology age estimates.

The Tsagaansuvarga Basin is part of a network of late Mesozoic grabens located in southwestern Mongolia (Figs 1 and 2), and interpreted to represent an episode of Late Jurassic-Early Cretaceous rifting [24]. This interpretation is based on seismic data showing Mesozoic strata thickening into normal faults, the abundance of bimodal volcanics, and regional correlations with well-known Jurassic-Cretaceous rift basins to the southeast in Mongolia and throughout eastern China (Fig 3) [24–30].

The basement rocks of the basin include Precambrian-aged metamorphic and igneous suites of multiple terranes and cratonal blocks, which were accreted during Neoproterozoic to Cambrian time [31,32]. These units are overlain by Paleozoic prerift rocks, which are mainly volcanic arc successions [33,34]. In the Tsagaansuvarga Basin, these Paleozoic units include Ordovician, Silurian, and Devonian sedimentary strata and volcanic units, as well as Carboniferous and Permian sedimentary rocks and intrusive complexes (Fig 1).

**Fig 4:**
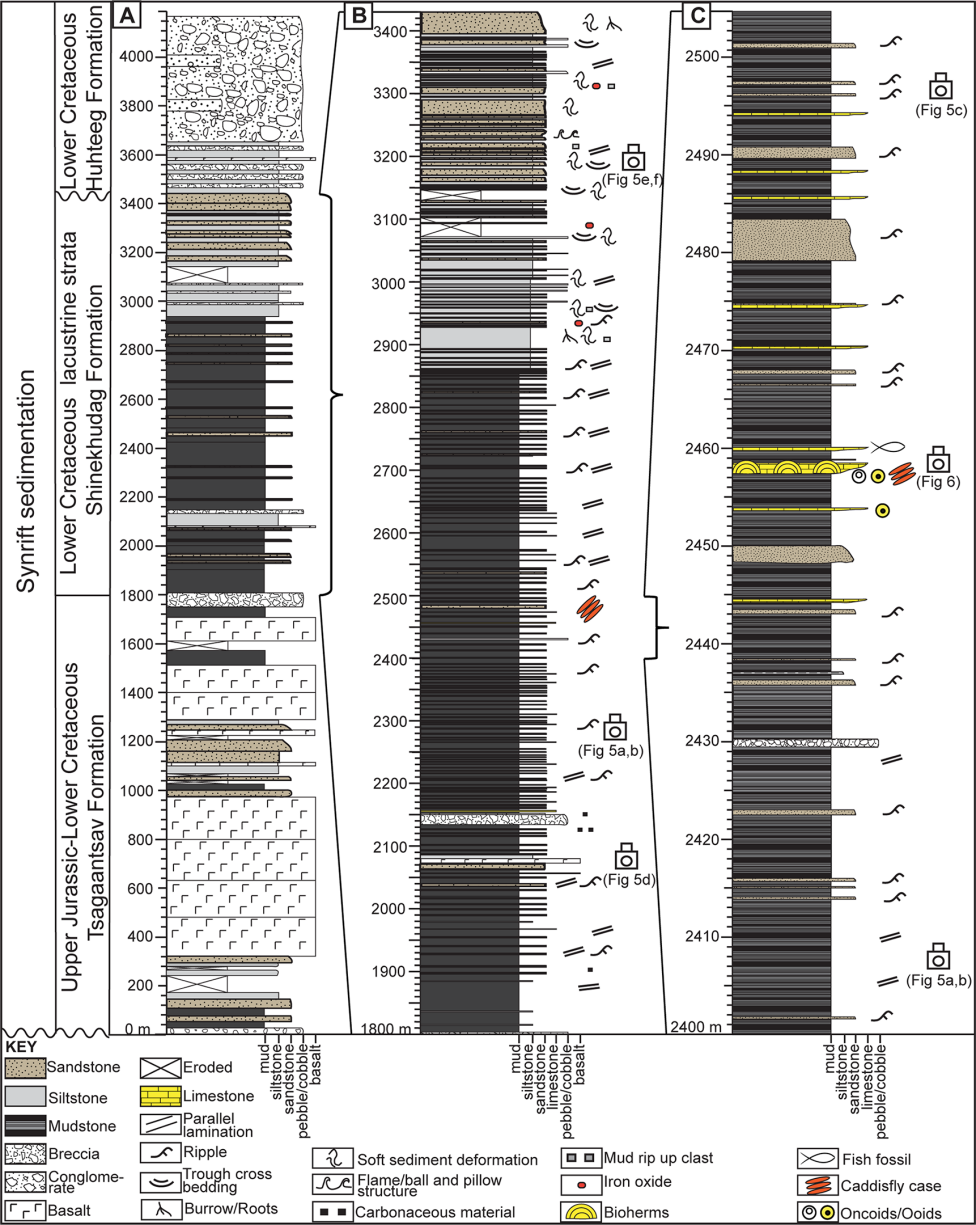
Tsagaansuvarga composite stratigraphic section. (A) Entire Tsagaansuvarga Basin fill from Upper Jurassic to Lower Cretaceous. (B) Main lacustrine facies of Tsagaansuvarga Basin (lower and upper interval). (C) Close view of microbial-caddisfly bioherm containing lower interval. Refer to the keys for lithologies and sedimentary structures.

Upper Jurassic-Lower Cretaceous basin fill includes more than 4000 m of nonmarine strata and volcanics, including significant volcanics in places. Palynomorph biostratigraphy suggests the basin fill, mapped as Tsagaantsav Formation at the base and overlain by the Shinekhudag Formation, ranges in age from Tithonian-Berriasian to Hauterivian (ca. 150–130 Ma; Waanders, written communication, 2011). This succession of basin fill is unconformably overlain by coarse clastic, proximal alluvial fan deposits of Huhteeg Formation (Fig 3). The composite stratigraphic section of the Tsagaansuvarga Basin (Fig 4) reveals an upward fining then upward coarsening succession of fluvial, marginal lacustrine, and distal lacustrine strata, investigated herein. We interpret this sedimentary evolution of the basin to be strongly linked to the tectonic processes controlling subsidence, with initial fault-controlled subsidence linked to upper crustal extension and subsequent thermal subsidence related to post rift cooling. This basin evolutionary trend is representative of many Mesozoic basins in Mongolia [27,30,35–37].

Most of the modern basin cover is mapped as Paleogene, Neogene, and Quaternary unconsolidated sediments (Figs 1 and 2). The east-west-trending, active Bogd fault system lies along the north boundary of the basin, and fault scarps from the 1957 M8.2 earthquake are still present to the northeast of the basin [38]. This Miocene to recent feature records sinistral transpression driven by far field deformation driven by the India-Asia collision, as recorded throughout the Gobi-Altai region (e.g., [37,39]).

## Materials and methods

The Petro Matad LLC issued the permission to conduct study on this site. Local field mapping of the Tsagaansuvarga Basin was conducted using a base of regional geological maps and satellite images. A compiled stratigraphic section of 4200 m was measured through the Jurassic-Cretaceous strata and thick igneous sills, primarily focusing on describing the lithology, grain size, sedimentary structures, and architecture of strata to interpret facies and depositional environments (Fig 2). Our main focus was on the lower interval of the Shinekhudag Formation (1800 m-2862 m; Fig 4) of finely laminated mudstone, as this has been previously identified as prospective hydrocarbon source rocks [25]. The putative microbial-caddisfly bioherms of primary interest for this study are mainly located in one discrete interval within this section.

A total of 22 hand samples were collected from these bioherms and all were analyzed for hand sample description (Table 1). Sixteen standard and large format petrographic thin sections were prepared from the hand samples for microscopic analysis. Three of them were partially stained in alizarine red-S dye for identification of carbonate minerals. A combination of Zeiss Axio Imager M2m petrographic scope, Zeiss Zen Blue Professional Software, and ImageJ 1.46r Software permitted qualitative grain and fossil size measurements, counts, and percentage estimation. All samples and thin sections are publicly available at the University of Utah.

**Table 1:** List of hand samples and classifications.

#	Sample Number	Classification
1	№1	boundstone
2	1B	boundstone
3	4BE210716_01A	packstone
4	4BE210716_1B	packstone
5	4BE210716_1C	oolitic packstone
6	4BH07011101	packstone
7	4BH07011102	packstone
8	4KC070111_02 (A)	boundstone
9	4KC070111_02 (B)	boundstone
10	4KC070111_02 (C)	packstone
11	4KC070111_02 (D)	packstone
12	4TM062511_01 (A)	packstone
13	4TM062511_01 (B)	packstone
14	4TM062511_01 (C)	packstone
15	4TM062511_02	packstone
16	4UK062811_01	packstone
17	4UK062811_01 (1B)	boundstone
18	4UK062811_03	packstone
19	AR	packstone
20	CD-16	boundstone
21	TA_01	boundstone
22	TA_02	packstone

## Results

### Part 1: Outcrop Geology

Mesozoic sedimentary basin fill deposits outcrop in a large area in the Tsagaansuvarga Basin (Figs 2 and 4). These Upper Jurassic-Lower Cretaceous strata lie unconformably on Paleozoic prerift successions, specifically Ordovician to Devonian intermediate grade metamorphosed (amphibolite phase), terrigenous sedimentary and volcanic units as well as their tuffs [33,34]. The stratigraphic relationship between the Mesozoic and Paleozoic is mainly mapped as an angular unconformity; however, the contact is not well exposed in the area (Figs 2 and 3).

The main lacustrine to marginal lacustrine facies of the late Mesozoic basin fill were originally mapped and separately designated as the Lower Cretaceous Shinekhudag Formation, which is dominated by fine-grained mudstone lithofacies, and the overlying Huhteeg Formation, dominated by coarser grained sandstone and conglomerate lithofacies (Figs 3 and 4) [33].

Prior to this study, the stratigraphic contact between the Lower Cretaceous Shinekhudag and Huhteeg Formations was placed at the facies change between more mudstone to sandstone dominated units, ~3093 m on our composite measured section (Fig 4A) [33,40]. This stratigraphic contact between the Shinekhudag and Huhteeg Formations has been reinterpreted to the 3448.5 m level, where a clearer depositional hiatus occurs, based on local geological mapping and depositional environment interpretations. A significant facies shift has been identified at this level from gray to tan colored, fine to coarse-grained sandstone beds to the red-green mottled siltstone beds (Fig 4A). The siltstone beds are generally massive, poorly cemented, and contains abundant coal fragments and coaly horizons. They are sporadically interbedded with thin (<5 cm) deeply weathered, blocky red mudstone intervals interpreted to be paleosols. This red-green siltstone interval transitions into clast to matrix supported conglomerate dominated strata around 3450 m. The conglomerate beds are predominantly red to purple colored, clast to matrix supported, granular to pebble sized, poorly sorted, and range in size up to 10 m thick (Fig 4A). Abundant trough cross beddings and clast imbrication can be found within these conglomerate beds.

Overall, these characteristics of the siltstone interval and overlying conglomerate dominated strata indicate alluvial depositional environments. We interpret the siltstone interval as a floodplain depositional environment, and the entire overlying conglomeratic succession as fluvial and alluvial fan deposits [41,42]. The facies juxtaposition between the floodplain siltstone and underlying sandstone beds at the 3448.5 m level suggests a period of aerial exposure, possibly an unconformity (Fig 4A). Similar facies contrasts as well as disconformable seismic reflection patterns were encountered in subsurface data collected from the adjacent Tugrug Basin (Fig 1B), and are interpreted as a minor synrift nonconformity [25].

Igneous sills from near the contact between Tsagaantsav and Shinekhudag Formations were dated as 117–109 Ma (Fig 3) [43]. Additionally, previous palynological studies identified occurrences of *Cicatricosisporites purbeckensis* (between 1526 and 1913 m level) and of *Vesperopsis fragilis* (between 1356 and 3302 m level) and both occurrences were estimated at Tithonian to Berriasian in age (Waanders, written communication, 2011). Bioherms, which are the main focus of this study, are located within the Shinekhudag Formation. Thus the following facies descriptions highlight this part of the synrift succession (Fig 4B and 4C).

#### Sedimentary facies description

The Lower Cretaceous Shinekhudag Formation of the Tsagaansuvarga Basin includes a variety of mudstone and sandstone dominated facies that together record overall evolution of this lake basin. The main lacustrine-marginal lacustrine intervals occur between 1800–3440 m on the general compiled stratigraphic section and these represent two distinct facies associations as described below, in stratigraphic order:

- Lower interval (1800–2862 m)

**Fig 5:**
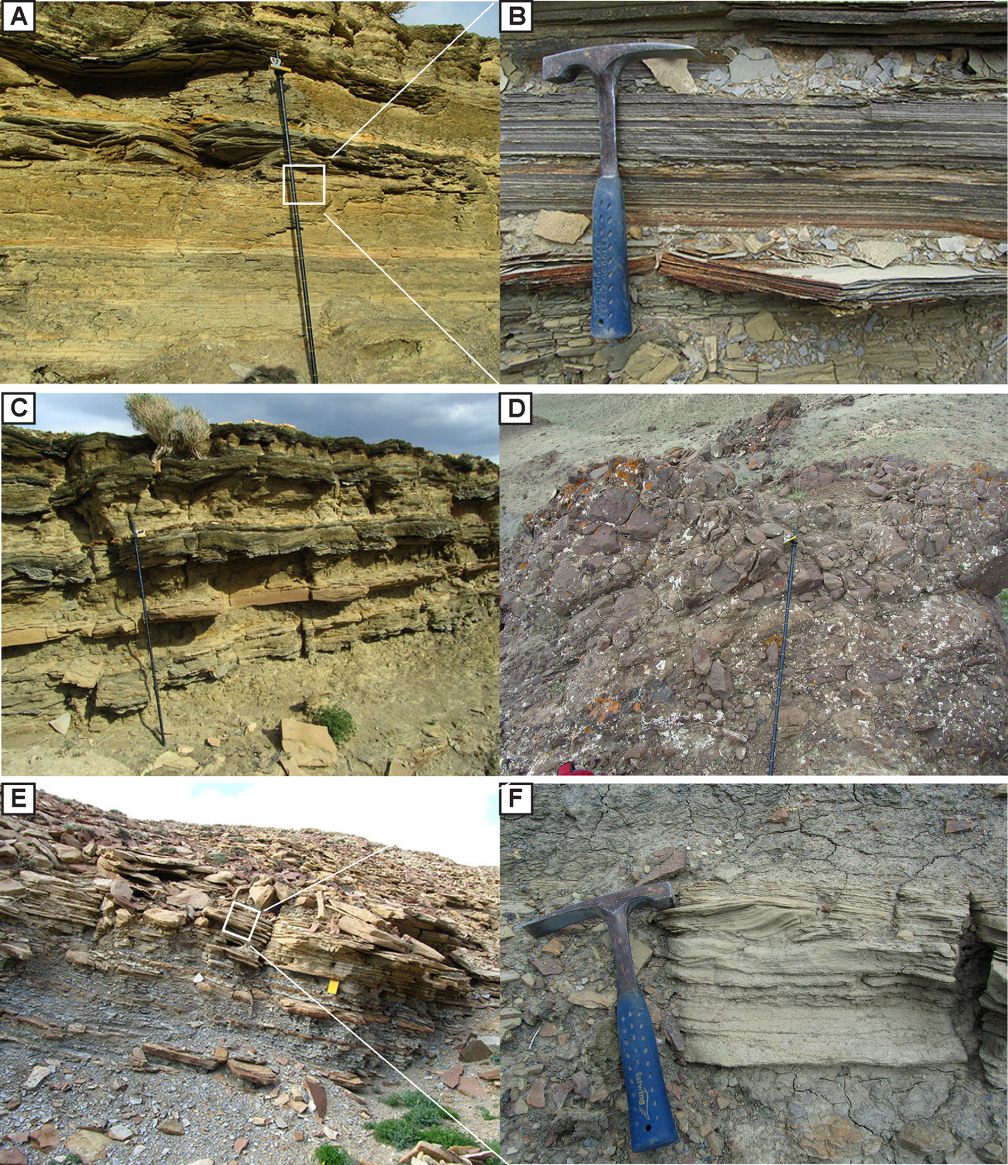
Photos of the main facies. (A) Laminated mudstone facies of the lower interval. (B) Close view of organic-rich, laminated mudstone. (C) Interbedded mudstone, tabular sandstone, siltstone, and marl facies. (D) Pillow lava of the lower interval. (E) Coarsening upward interbedded sandstone, siltstone, and mudstone facies within the upper interval. (F) Climbing ripples of the sandstone facies within the upper interval.

This interval includes interbedded mudstone, siltstone, sandstone, marlstone, and conglomerate that has been inflated by thick sills of basalt (Fig 4B). The fine-grained facies range from dark, mm to cm-scale, fine laminated mudstone and fissile shale to more massive decimeter-scale bedded mudstones and siltstones (Fig 5A and 5B). The fine mm to cm-scale laminated mudstones and fissile shales are often organic rich, with sporadic plant fragments. Prior to this study, numerous mudstone samples were analyzed from this lower interval for source rock potential. Total organic carbon (TOC) results revealed that the mudstone facies are indeed oil shales, rich in organic carbon, with TOC values ranging from 1.60% to 10.26% (average 5% by weight) [43].

The mudstone and siltstone units of the lower interval are commonly interbedded with fine to coarse-grained, normally-graded, tabular sandstone as well as marlstone beds (Fig 5C). The sandstone beds are up to 2 m thick, tan-brown colored, and contain abundant ripple cross stratification as well as planar laminations. The marlstone beds are 0.2–2.0 m thick, tan-brown to pink colored, typically have planar laminations, are laterally continuous, and are likely dolomitic in composition as they are weakly soluble to insoluble by dilute hydrochloric acid.

**Fig 6:**
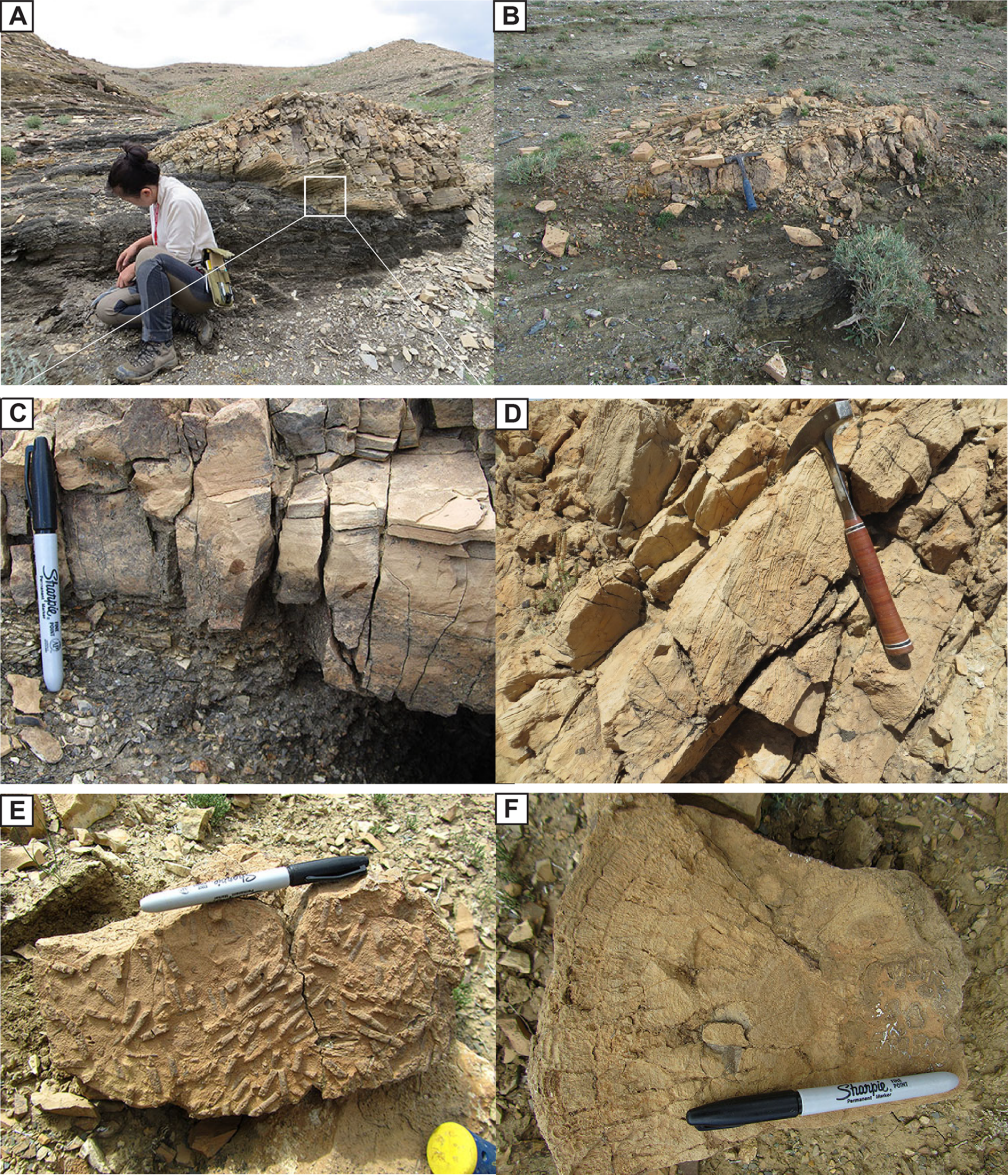
Photos of the bioherms. (A) Mound-shaped bioherm bounded by laminated, organic-rich mudstone. (B) Mound-shaped bioherm bounded by laminated mudstone. (C) Contact between bioherm and underlying mudstone. (D) Wavy laminated structure of the bioherm. (E) Tube-shaped caddisfly cases on the weathered surface of bioherm. (F) Fine laminated internal structure of the bioherm.

At the 2458 m level, there are multiple distinctive, resistant, tan-brown, convex upward, mound-shaped, patch reef-like carbonate bodies identified, which are bounded by discrete stratigraphic interval between thick organic-rich mudstones (Fig 6). The contact between the mounds and the underlying oil shales is abrupt with no indication of a subaerial exposure (Fig 6A, 6B and 6C). The diameter of individual mounds is 0.5–2.0 m and their thickness ranges from 1–5 m. The main cluster of these mounds is concentrated within one stratigraphic interval, with individual mounds isolated from each other laterally by 30–40 m. The interval can be traced for more than 1 km along strike (Fig 2). Additionally, there are a few mounds discovered in a fault sliver, approximately 2100 m east from the main cluster of carbonate mounds (Fig 2); based on geological mapping, the fault-sliver mounds are likely part of the same overall stratigraphic interval as the main clusters. Internally, mounds are characterized by mm-scale wavy laminations and they contain ooids, oncoids, and abundant colonies of tube-shaped, cylindrical structures interpreted as insect cases due to their similarity to others that have been previously reported from the Eocene of North America and elsewhere (Fig 6D, 6E and 6F) [13,19]. These insect cases can make up to 40% of the volume of a single mound (Fig 6E).

The coarsest grained facies of the lower interval are characterized by granular to pebble sized, moderately to poorly sorted, matrix supported conglomerate beds, up to 3 m thick. The conglomerate beds characteristically lack sedimentary structures, except rare poorly defined planar cross beds. Between 2133 m to 2150.5 m, there is a megabreccia with chaotic soft sediment deformation, which contains dismembered m-scale sandstone, mudstone, and dolomite boulders.

The volcanic components within this interval are mainly characterized by a single bed with flow texture and thin potential ash beds that are poorly preserved in outcrop (Fig 5D). A 4 m-thick basalt unit with pillow texture is found near the base of the lower interval, located at approximately 2075 m on our measured section (Fig 4B). The basalt bed has porphyry texture with plagioclase phenocrysts along with other fine mafic minerals.

- Upper interval (2862 m-3440 m)

This interval is generally sandier in comparison to the lower interval. Overall, the interval consists of mudstone, siltstone, sandstone, and conglomerate (Fig 4B). The fine-grained facies range from gray to dark gray colored, fine laminated mudstone to more massive siltstone beds. Horizontal burrowing and soft sediment deformation are pervasive throughout the mudstone and siltstone facies. The mudstone beds are siltier in composition and contain more carbonaceous material compared to the lower interval. The fossil content of this interval is generally sparse except invertebrates such as ostracods and gastropods found within the mudstone and siltstone units, as well as unidentifiable, reworked plant fragments found within the sandstone units.

The mudstone and siltstone facies are commonly interbedded with tan-brown colored, fine to coarse-grained sandstone beds. These units are typically normally-graded and moderately sorted with abundant trough cross bedding, climbing ripples, and soft sediment deformation as well as mud rip-up clasts. They commonly form lenticular bodies with erosive bases, up to 8 m thick and 12 m wide (Fig 5E and 5F). The sandstone units typically overlie and locally scour into mudstone and siltstone units and a few possible root casts are present at the 3381.5 m level. Overall, the thickness of the sandstone beds tends to increase from dm-scale to m-scale up section.

The coarsest grained facies of the upper interval consists of up to 10 m successions of gray colored, granular to pebble-cobble sized conglomerate beds containing quartzite, chert, argillite, sandstone, siltstone, basalt, and andesite clasts. These conglomerate beds are characteristically tabular to lenticular with sharp bases, and mainly overlie siltstone and mudstone beds. The tabular conglomerate beds tend to be matrix supported, massive, and poorly sorted, while the lenticular conglomerate beds are mainly clast supported and have trough cross beds as well as strong clast imbrications.

#### Sedimentary facies interpretation

The lower and upper intervals of the Lower Cretaceous Shinekhudag Formation reflect two distinct lacustrine facies associations. Here we used the Carroll and Bohacs’ (2000) [44] three end-member lacustrine facies association classification to describe and interpret each.

The lower interval (1800 m-2862 m) (Fig 4B and 4C) is assigned to the fluctuating-profundal facies association. The fine-grained facies of this interval includes finely laminated, organic rich mudstone, siltstone, and dolomitic marl beds with terrestrial and aquatic organic matter. These predominantly fine-grained, mudstone, and siltstone beds are consistent with the distal deep lake sediments resulted from sediment suspension settling and traction deposition, thus we interpret them as a profundal offshore lake environment [45–47]. The fine, mm to cm-scale lamination and the high concentration of organic matter (average 5% TOC) preserved in this offshore mudstone facies is highly diagnostic of the anoxic hypolimnion part of a stratified lake environment [44,48,49]. The laterally homogenous, laminated marl beds interbedded with the mudstone and siltstone are interpreted as chemical sediments precipitated from Ca/Mg and HCO_3_ bicarbonate rich lake water, within the clastic-input-starved offshore lake environment [50].

The mound-shaped carbonate units that are concentrated within a thick horizon of oil shale at 2458 m level (Fig 4C) are quite enigmatic in terms of their depositional processes and relationships with the bounding facies. Their physical appearance, internal sedimentary structure, and relationship with the surrounding lithofacies suggested that these are bioherms, defined by Klement and others (1967) [51] as “a massive mound shaped structure, that is discordant in relationship with the surrounding facies with different lithologic types”. Co-occurrence of the ooids and oncoids in these bioherms indicate a shallow water setting with wave modification, while the diagnostic traces of insects, the caddisfly cases, described further in the next section, indicate a well-oxygenated, nutrient rich environment, perhaps the littoral zone of the lake [23,48,49]. Although perplexing, the presence of shallow/fresh water bioherms bound above and below by profundal lake facies has been documented in other modern and ancient examples (e.g., modern Great Salt Lake, Eocene Green River Formation) [14,19,52], and these relationships are discussed further below.

The tabular sandstone facies in this interval (1800 m-2862 m) are consistent with distal turbidite beds, resembling a partial Bouma sequence that possibly resulted from a hyperpycnal plume, perhaps in a subaqueous prodelta environment (Fig 5C) [49,53]. The clast- to matrix-supported, poorly sorted conglomerate beds are interpreted here as mass transport deposits including high-density turbidites and debris flows, possibly in a subaqueous fan delta environment [54,55]. The massive, matrix-supported, ~18 m thick megabreccia bed is interpreted as a large-scale debris flow deposit within a subaqueous landslide, perhaps resulting from a seismic event. The basaltic pillow lava flow (Fig 5D) and cm-scale potential ash beds at the base of the section are possibly the result of a subaqueous volcanic extrusion: an indication of syndepositional magmatism.

Thus, we interpret the lower interval of the Shinekhudag Formation as mainly offshore lake sediments characterized by the fluctuating-profundal facies association (balance-filled lake basin type) [45,46,49]. Possible distal fan delta beds encased in distal lake facies can be attributed to steep relief of the lake margin, which is a common feature in rift-associated, deep lakes [56]. The high topography of the lake margin possibly accelerated progradation of these fan delta beds, and they grade rapidly into offshore turbidites [57]. Regional seismic sections from previous studies showed significant basin fill thickening away from the basin ramp margin [25,40], supporting the interpretation of a deep, stratified lake.

The upper interval (2862 m-3440 m) (Fig 4B) of the Lower Cretaceous Shinekhudag Formation is assigned to the fluvial-lacustrine facies association, a combination of both offshore lake deposits, marginal proximal deltaic, and fluvial sediments. The lower portion of this interval (~2862 to 3148 m) is transitional with the underlying fluctuating profundal facies association; it is characterized by fine-grained mudstone to siltstone dominated facies and gradually coarsens upward to sandstone dominated facies (~3148 to 3430 m; Fig 4B).

The fine-grained facies of this upper interval are dominantly interbedded mudstone and siltstone beds characterized by tabular, laterally continuous beds with fine laminations as well as soft sediment deformation and ripple cross stratification. Compared to the underlying section, the upper interval contains more clastic-rich, silty mudstone with fewer laminated oil shales, and dolomite beds (Fig 5E). In general, deposition of these units is dominated by both suspension settling and bedload transport, in a low-energy distal lacustrine environment. However, the internal sedimentary structures coupled with the vertical to sub-vertical burrowing within the siltstone units suggest a slightly higher energy environment than comparable facies in the lower interval. Thus, we interpret the fine-grained facies as a distal offshore lake deposit that was at least temporarily stratified but also periodically dominated by a higher energy subaqueous prodeltaic or fan delta environments [55].

Sandstone beds of the upper interval represent fluvial channel fill, as indicated by their lenticular shapes, erosive bases, normal grading, common mudstone rip-up clasts, and both trough and planar cross bedding (Fig 5E and 5F). The internal sedimentary structures and possible root casts are an indication of a high-energy fluvial-deltaic complex, or a delta front or shoreface depositional environment [42,58]. In comparison to the channelized sandstone units, the tabular, massive conglomerate beds within the mudstone and siltstone facies (e.g., 2970 m to 3070 m) are more consistent with the high-density turbidites and debris flow deposits, possibly in a fan delta environment (Fig 4B) [41,53,54]. Similar to the lower interval, these fan delta beds are possibly the subaqueous portion of an alluvial fan environment along a steep margin of the lake [56,57].

The large-scale gradual transition of the basin fill lacustrine facies association from fluctuating-profundal (lower interval) to fluvial-lacustrine (upper interval) reveals progressive evolution of the lake basin as well as gradual shifting of the lake shoreline. The transition of the facies association is controlled by relative balance of sediment and water input versus potential accommodation (Fig 4B) [45,46].

### Part 2: Description of bioherms

The detailed morphologies of the Lower Cretaceous Shinekhudag Formation bioherms (measured at ~2458 m level; Figs 2, 4C and 6) are described at three levels following the recommendations of Shapiro (2000) [59]. Macrostructures include bioherm geometry, dimensions, and outcrop expressions, as described under facies descriptions above. Mesostructural-level description includes the detailed studies of the internal bioherm fabrics in hand samples, and petrographic observations are the microstructural level of investigation.

#### Mesostructure

Twenty-two hand samples were classified following Dunham (1962) [60]. The majority (14) of the hand samples are packstones containing caddisfly cases as well as ooids and oncoids: they are grain-supported with both micrite matrix and calcite cement. Seven samples are boundstones, composed of mm-scale laminated carbonates, caddisfly cases, and other grains. One remaining sample is purely an oolitic packstone with no caddisfly cases, nor any microlaminated carbonate (Table 1).

**Fig 7:**
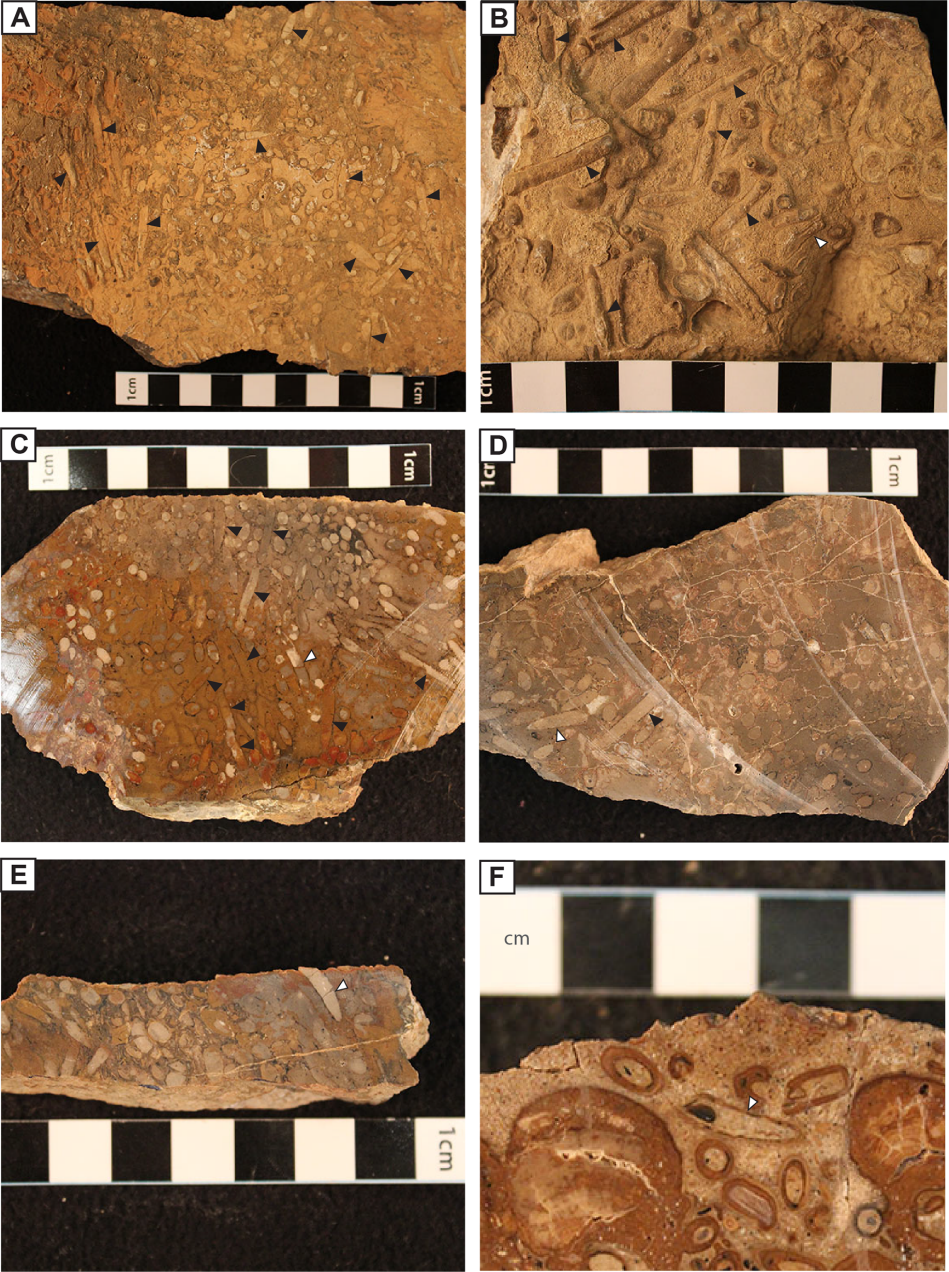
Caddisfly cases on the hand sample photos. (A) Straight (black arrow) caddisfly cases on the hand sample. (B) Straight (black arrow and curved (white arrow) caddisfly cases on the hand samples. (C) Straight (black arrow) and curved (white arrow) caddisfly cases on the polished sample. (D) Straight (black arrow) and curved (white arrow) caddisfly cases on the polished sample. (E) Curved (white arrow) caddisfly case on the polished sample. (F) Curved (white arrow) caddisfly case on the polished sample.

Caddisfly cases form up to 75% of the packstone sample volume, ranging from loosely to densely packed, along with other grains including ooids, oncolites, plant fragments, and rare clastic grains (Fig 7). The caddisfly cases can be recognized easily on the eroded surface of the hand samples, displaying a variety of morphologies (Fig 7). The case shape varies with position relative to the eroded surface: they are round or circular shaped when perpendicular, elliptical when positioned obliquely and cylindrical tube-shaped when they are parallel.

**Fig 8:**
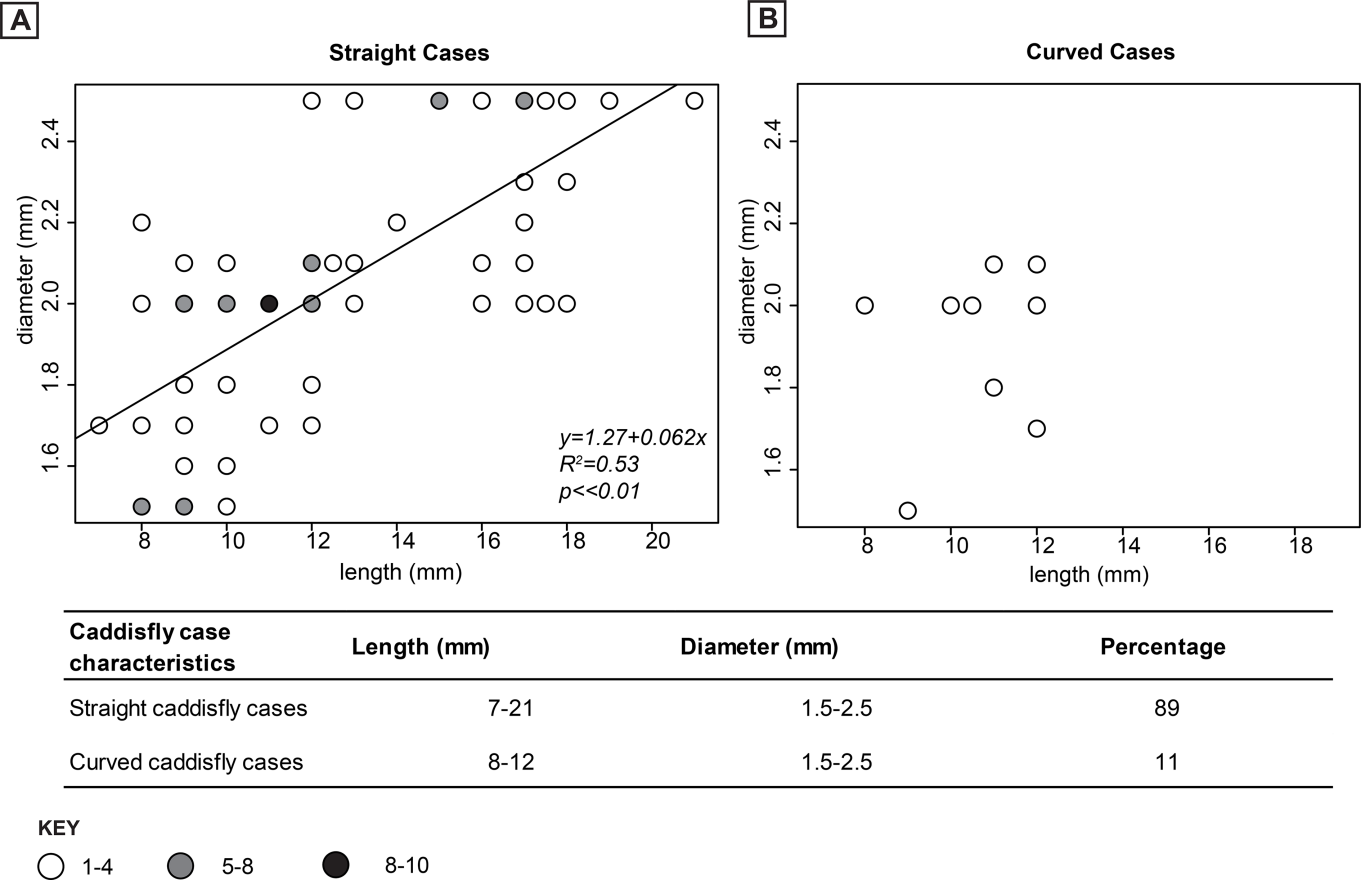
Caddisfly case size variations. (A) Size distribution of the straight caddisfly cases. (B) Size distribution of the curved caddisfly cases. (1–10)- Number of caddisfly cases.

**Fig 9:**
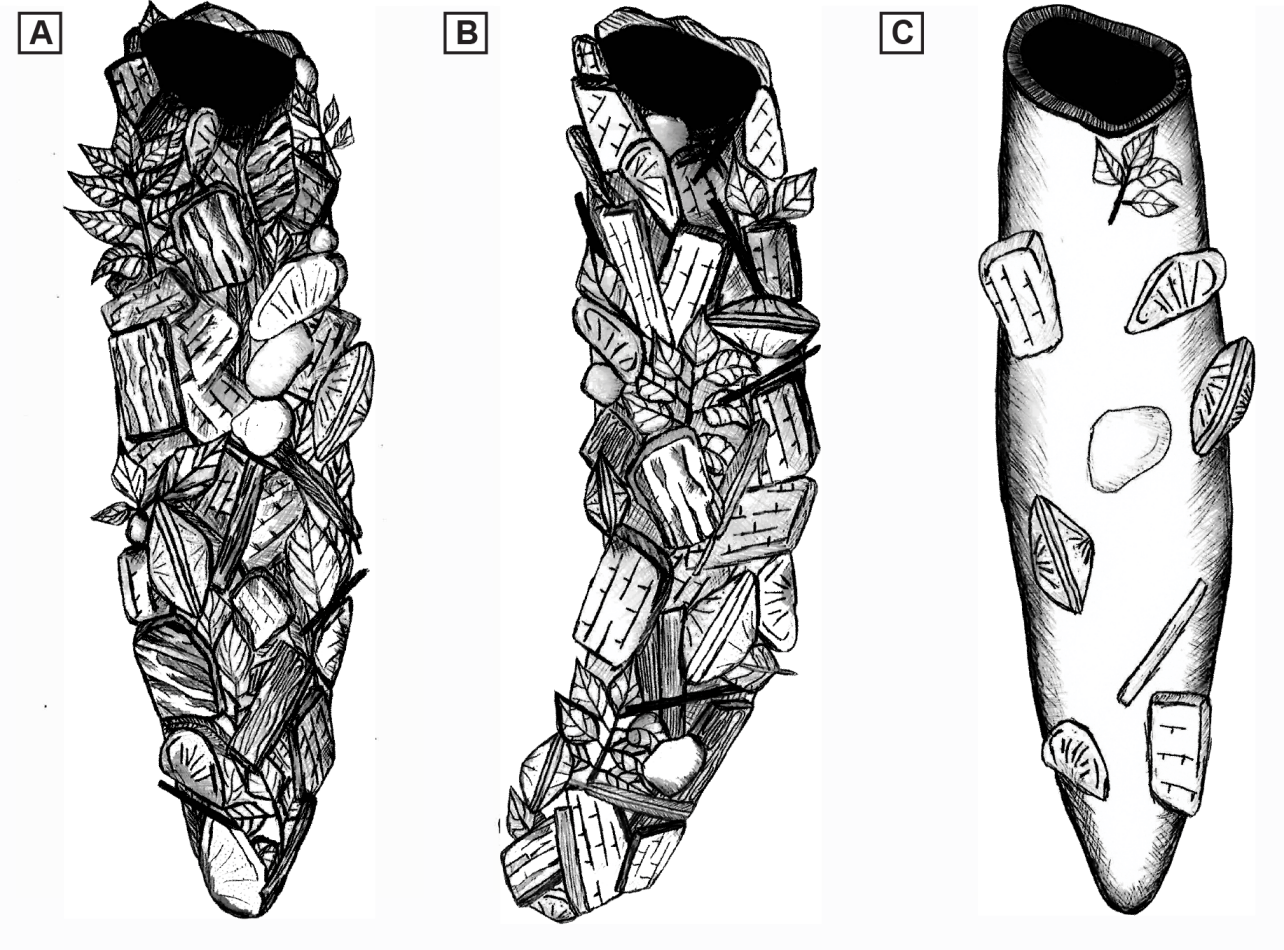
Caddisfly case reconstruction. (A) Straight caddisfly case. (B) Curved J-shaped caddisfly case. (C) Straight caddisfly case. Cases are lined with plant fragments, ostracod valves, mica, and carbonate rock fragments. Note: Caddisfly case particles are for illustration purpose, not to scale.

The sizes and shapes of the caddisfly cases are nonuniform on the hand samples. We measure a total of 115 caddisfly case sizes from their caudal to cephalic ends on the hand samples. Based on these measurements their lengths and internal diameters have progressively increasing trend, ranging between 7–21 mm and 1.5–2.5 mm, respectively (Fig 8). The majority of the caddisfly cases are straight with no curvature along their long axes in most examples (Fig 7); however, some are occasionally slightly curved along their long axes forming a “J” shape (Fig 7). Based on their shape differences, we defined two types of caddisfly case distributions (Figs 7,8 and 9).

- Straight caddisfly cases

The majority of the caddisfly cases documented from the hand samples have straight cylindrical tube shapes (Fig 7A, 7B, 7C and 7D). The lengths and internal tube diameters of this type of cases vary, with their length ranging between 7–21 mm, and diameter ranging between 1.5–2.5 mm (Fig 8). They are slightly tapered to their posterior ends and they are mostly visibly open to their anterior ends. These cases are mainly filled with calcite cement and can rarely be empty (Fig 7A, 7B, 7C and 7D). The cases are lined with about 1 mm thick, dark brown to black colored organic carbonaceous materials interpreted as plant fragments as well as other minor grains (Fig 7A, 7B, 7C and 7D). They occur with no to very minor degree of alignment and can be found with or without the other types of cases (Fig 7A, 7B, 7C and 7D).

- Curved caddisfly cases

The curved caddisfly cases are rare in comparison to the straight cases. These cases are slightly curved along the long axis forming curved J-shapes (Fig 7B, 7C, 7D, 7E and 7F). The lengths of the curved caddisfly cases range between 8–12 mm and diameters range between 1.5–2.1 mm (Fig 8). They are slightly tapered to their posterior ends, but the anterior ends were not described due to their rareness. Abundance of this type of cases may be underestimated due to their similar appearance to the straight caddisfly cases in cross section and oblique view (Fig 7B, 7C, 7D, 7E and 7F).These cases are commonly filled with carbonate mud and lined with thin calcite as well as dark colored plant fragments (Fig 7B, 7C, 7D, 7E and 7F). The construction of these cases is clarified by thin section description, below.

#### Microstructure

**Fig 10:**
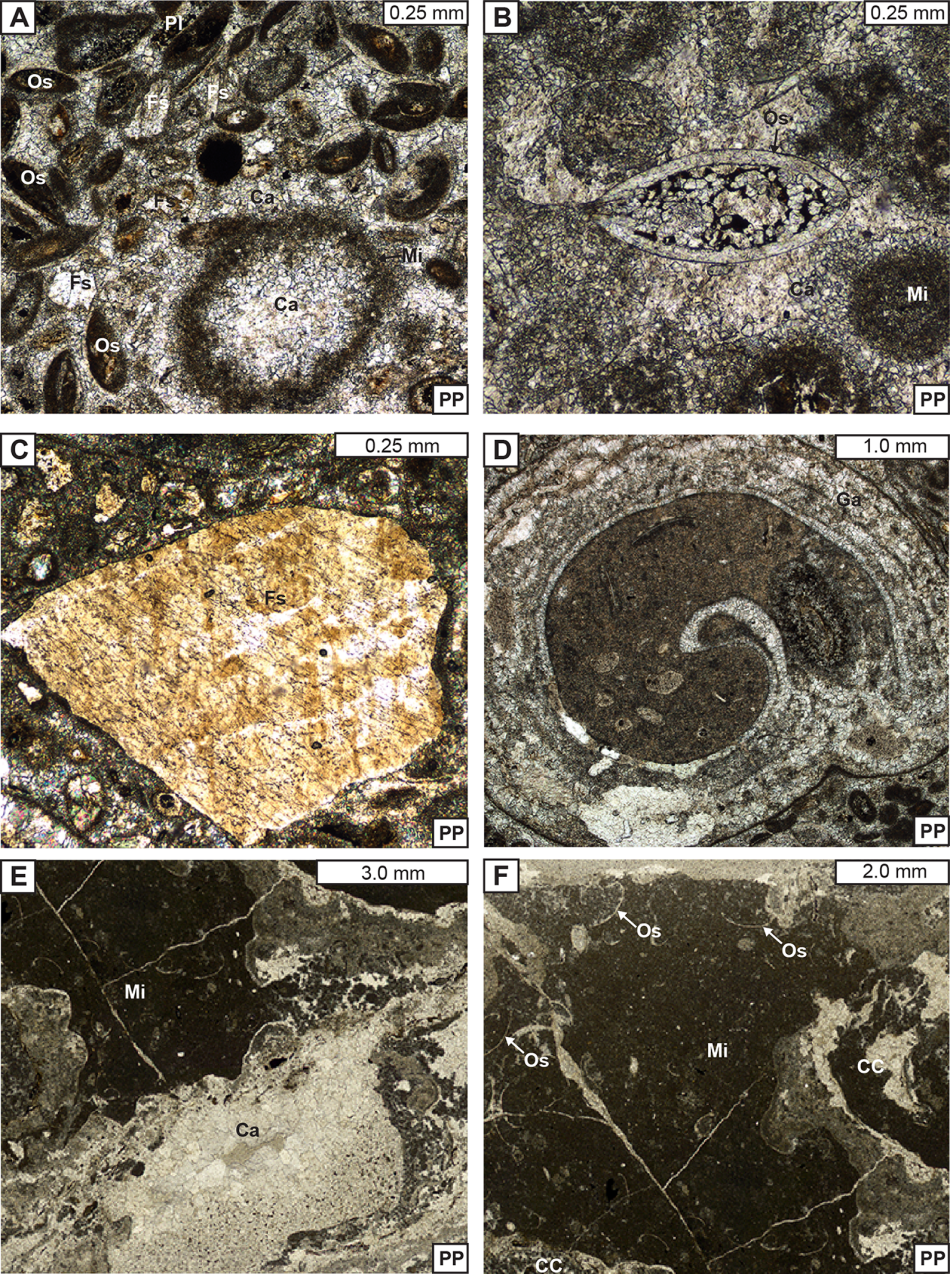
Photomicrographs of the packstone. (A) Allochems of the packstone cemented by fine-medium sparry calcite. (B) Intact ostracod allochem. (C) Detrital feldspar. (D) Gastropod. (E) Contast showing massive micrite matrix and various-sized calcite cement of packstone. (F) Massive micrite matrix. CC-caddisfly case; Fs-feldspar; Os-ostracod; Ga-gastropod; Ca-calcite; Mi-micrite; Pl-plant fragment.

**Fig 11:**
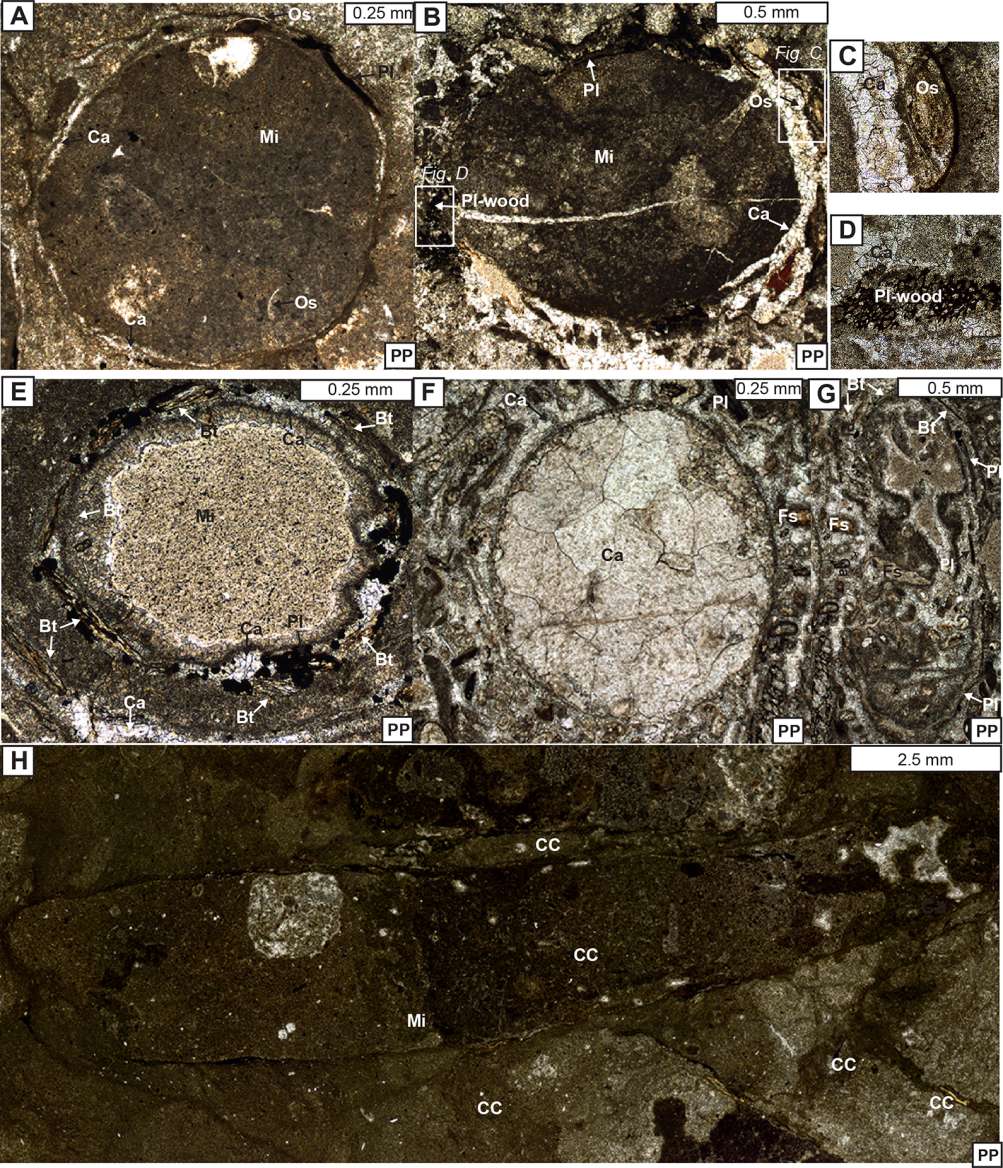
Photomicrographs of the caddisfly cases. (A) Caddisfly case in cross section. (B) Caddisfly case in cross section. (C) Close view of an ostracod case particle. (D) Close view of a plant-wood fragment. (E) Caddisfly case in cross section with biotite fragments. (F) Caddisfly case in cross section, filled with blocky calcite spar cement. (G) Oblique view of caddisfly case. (H) Caddisfly case in longitudinal section. Ca-calcite; Mi-micrrite; Pl-plant fragment; Os-ostracod; Bt-biotite; Fs-feldspar; CC-caddisfly case.

**Fig 12:**
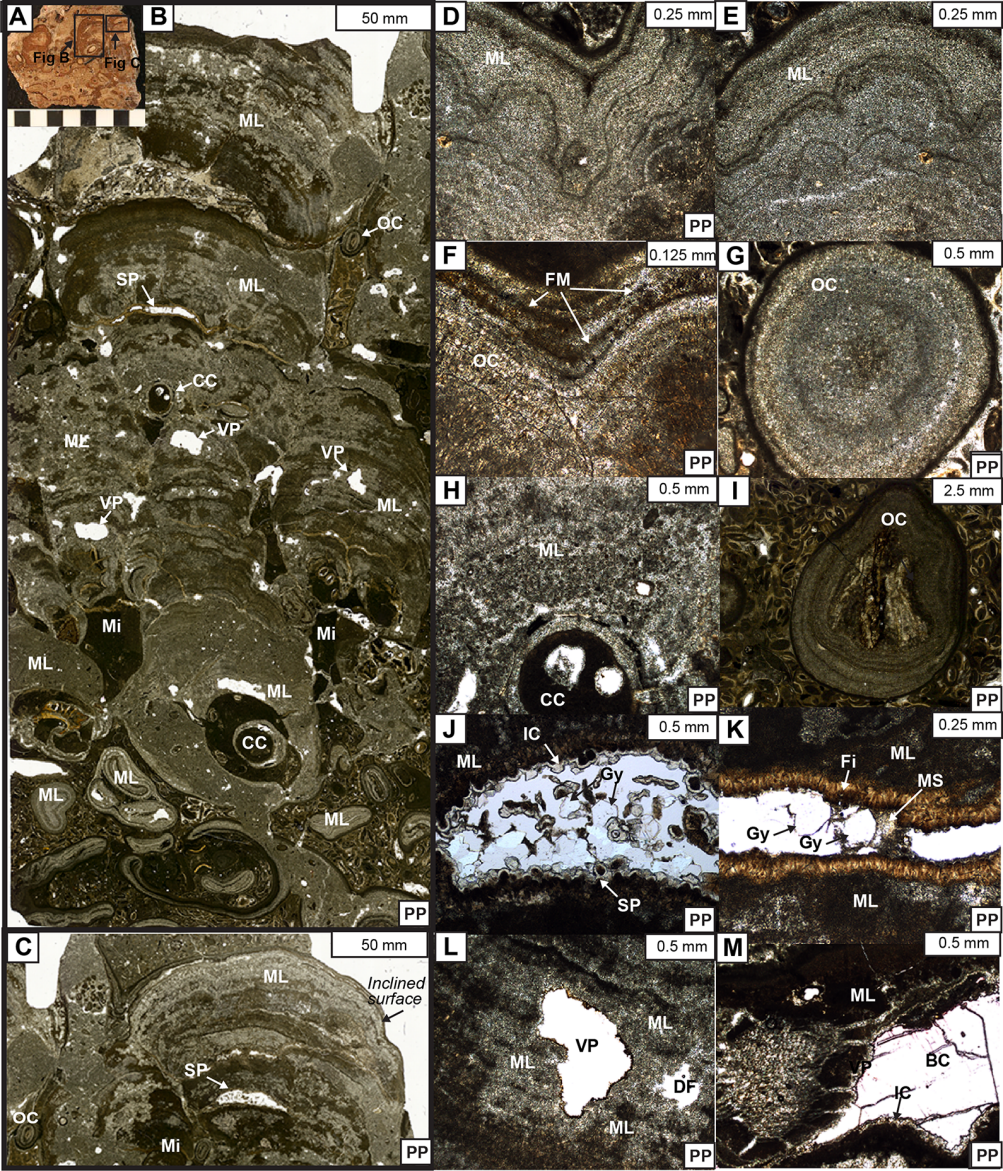
Photomicrographs of the microlaminated micrites. (A) Hand sample photo of the boundstone. (B) Microlaminated micrite forming finger-like columnar fabrics. (C) Cone shaped microlamination. (D) Crinkled, wavy lamination with inclined surface. (E) Crinkled, wavy lamination. (F) Flocculated micrite grain trapped/bound between laminations. (G) Sphere shaped oncoidal fabric in cross section. (H) Caddisfly case bound by fine laminations. (I) Sphere oncoidal fabric with a plant nuclei. (J) Shelter porosity and its associated cements. (K) Shelter porosity and its associated cements. (K) Shelter porosity and its associated cements. (L) Vugular porosity. (M) Vug porosity and its associated cements. CC-caddisfly case; ML- microlaminated micrite; Mi-micrite matrix; SP- shelter porosity; VP- vugular porosity; OC-oncoid; FM- flocculated micrite; MS- granular microspar; IC-isopachous calcite; BC- blocky calcite; Gy- gypsum; Fi- fibrious calcite.

Sixteen standard and large format thin sections were analyzed for microstructure description; three of them were stained on ½ of the section with alizarine red S-dye for improved identification of carbonate mineralogy. All samples display original depositional fabrics with little impact of diagenesis except cementation, as described below. The majority (10) of the thin sections were prepared from the packstones that are composed of caddisfly cases (~75%) and various allochems (~25%). These samples show microscopic details of the main skeletal and non-skeletal grains of the packstone along with the fine-grained matrix, and cement (Figs 10 and 11). The remaining six thin sections are from the boundstones that are composed of fine laminated carbonate (~60%), caddisfly cases (~10%), and various allochems (~30%). These samples show clearly defined mm-scale microlaminated fabrics as well as association of both microlaminates and other grains including the caddisfly cases (Fig 12). We describe detailed depositional and diagenetic microfacies of the packstones and boundstones separately due to their compositional differences.

**Depositional microfacies of the packstones:** Here, we mainly focus on petrographic characteristics of the packstone grains and matrices. The grains of the packstones are predominantly composed of caddisfly cases and lesser amount of ooids, oncoids, plant fragments, ostracod valves, carbonate grains, as well as sporadic feldspar and biotite grains (Figs 10 and 11). The caddisfly cases and other allochems are packed together by massive microcrystalline micritic matrix (Fig 10E and 10F). These matrices are mainly dolomitic in composition, as they show no staining from the alizarine red-S dye [61,62].

In thin section, caddisfly cases show tight to loosely packed, aligned to non-aligned caddisfly cases with some degree to no orientation pattern. Similar to the hand samples, caddisfly cases also display a variety of shapes in thin section: circles when they are cut perpendicular, ellipses when they are cut oblique, and long elongated ovals when they are cut parallel to their long axes (Fig 11). These cases show a variety of materials incorporated for their case construction as discussed below.

**Caddisfly case architecture:** Approximately 90% of the caddisfly cases are nearly completely lined with diverse types of debris which cover most of their exteriors (Fig 11A, 11B, 11C, 11D, 11E, 11G and 11H). The remaining 10% of the caddisfly cases are weakly lined with a few to no debris attached to the case exteriors (Fig 11F). The caddisfly cases are mostly covered with an abundant amount of dark brown to black colored, almost opaque, thin, and elongated grains of plant fragments, a lesser amount of ostracod valves, subrounded carbonate grains, and occasionally fine to very fine-grained detrital clastic grains such as biotite and feldspar (Fig 11, Table 2). Plant fragments are the most abundant type of material in the case exteriors, and about 30% of the plant fragments were originally wood pieces with diagnostic cellular structures (Fig 11B and 11D). The remaining 70% of the plant fragment has no identifiable structure (Fig 11A, 11G and 11H). The average length of plant fragments found in the caddisfly cases (n = 108) is 0.66 mm and the average thickness is 0.1 mm. Rarely, the woody materials reach up to 1.97 mm in length and 0.53 mm in thickness. The next most common type of the caddisfly case construction materials are the ostracod single and intact valves (n = 30), with average lengths of 0.58 mm and average thicknesses of 0.06 mm (Fig 11B and 11C, Table 2). Additionally, elongated, dark-brown colored, pleochroich biotite grains (n = 12) form a minor component of case particles, with average lengths of 0.26 mm, average widths of 0.04 mm, and rare feldspar grains (n = 8) with average lengths of 0.37 mm and average thicknesses of 0.20 mm (Fig 11E and 11G, Table 2).

**Table 2:** Thin section clast counts of the caddisfly case construction materials.

Type of construction materials	Number of counts	Percentage %
plant fragment	108	68
ostracod	30	19
biotite	12	8
feldspar	6	4
carbonate rock fragment	4	3
Total	160	100

**Diagenetic microfacies of the packstones:** The main diagenetic microfacies criteria are cements, neomorphic alterations, dissolution features, and fracturing [63–66]. However, no alteration, dissolution, and fracturing features associated with the packstones were documented, thus cementation is the main point of description. The main types of porosities of packstones include the interparticle porosities observed between caddisfly cases and other allochems as well as the intraparticle porosities observed within the interiors of caddisfly cases (Figs 10 and 11) [67]. Both porosity types are filled with various-sized sparry calcite cements. The original interparticle pore spaces are chiefly cemented by the fine to medium grained calcite spars (Figs 10A, 10B, 11F and 11G). The intraparticle pore space of the interior of the caddisfly cases are filled with coarse-grained blocky calcite cements with distinct crystal boundaries (Fig 11F). These cements are presumably dolomitic in composition, as they show no staining from the alizarine red-S dye [61,62], however in thin section (described below) no clear evidence of primary dolomite precipitation was observed.

**Depositional microfacies of the boundstones:** The main focus of the boundstone microfacies is mm-scale microlaminated micrite. In thin section, the microlaminated micrite fabrics are mainly defined by irregular alterations of wavy, crinkled to semi-crinkled laminations of dark green-brown colored micrite to light brown colored sparry calcite layers (Fig 12). These laminations formed relatively flat to finger-like, microdigitate columns (4–12 mm in width and 6–17 mm in height) and cones with high angle inclinations as well as sphere-shaped oncoid fabrics (Fig 12B, 12C, 12G and 12I). The average diameter of oncoids is ~4 mm and composed of layers of wavy, semi-crinkly microlaminations (Fig 12G and 12I). The microlaminations tend to drape and pinch out towards the steeply inclined sides of the columns and cones (Fig 12B, 12C, 12D, 12E and 12F). These microlaminates are commonly associated with other grains during their sedimentation, such as binding and trapping particles between layers, growing around caddisfly cases, and reworking detrital grains into nucleation sites for oncoids (Fig 12B, 12H, 12F and 12I). Occasionally, dark brown to almost black colored, very fine-grained, sphere shaped particles are bound between the layers of microlaminations, especially on the high angle, inclined surfaces. These particles may result from carbonate mud flocculations (Fig 12F) [68]. Also, oval to circular-shaped caddisfly cases are documented within the interior of these microlaminates (Fig 12B and 12H). Additionally, as with the lined casings, various types of grains (e.g., ostracod, plant fragments, and detrital feldspar grains) are accumulated between the dome, cone, and sphere-shaped microlaminated micrite growths (Fig 12B).

**Diagenetic microfacies of the boundstones:** The cementing and dissolution history was our main descriptive focus for the boundstone diagenetic microfacies description. The main types of porosities of the boundstones include primary shelter porosities associated with the microlaminates, and secondary vugular porosities associated with the dissolution fabrics (Fig 12B, 12C, 12J, 12K, 12L and 12M) [67].

Primary shelter porosities between the microlaminates are mostly half dome shaped and filled with 2–3 stages of syndepositional to early diagenetic cements in the following order: (1) isopachous calcite cement & fibrous calcite; (2) gypsum or (1) isopachous & fibrous calcite; (2) gypsum; (3) granular microspar (Fig 12C, 12J and 12K). The first stage of the cement between shelter porosities includes needle-like, fine to medium-crystalline fibrous calcite as well as subhedral, medium-crystalline isopachous calcite cements. They fully line interior of the pore spaces with 0.05 to 0.1 mm thicknesses (Fig 12J and 12K). The second stage of the cement between the shelter porosities is characterized by euhedral to subhedral, low-relief, and platy crystals of gypsum, with diameters ranging 0.06 to 0.25 mm (Fig 12J and 12K). Granular calcite microspars, the last stage of the cement, are crystallized between the preceding calcite and gypsum cements (Fig 12K).

Secondary porosities found within the thin sections are often associated with the dissolution. They are predominantly characterized by various sized, irregular vug shapes, with diameters ranging from 0.09 to 2.0 mm. (Fig 12B, 12L and 12M). These secondary pore spaces are partially rimmed with a single stage of the isopachous calcite cements. Occasionally, they are fully filled with isopachous, as well as blocky calcite cements (Fig 12L and 12M).

Based on the staining method, the mineral compositions of boundstones are dominantly composed of dolomite for about 60% and calcite for about 40%. These dolomite cements are predominantly found within the primary pore spaces (Fig 12M) [61,62]. However, in thin sections, there is no clear evidence of primary dolomite fabrics [69,70]. Therefore, arguably these dolomite cements are likely not syndepositional, instead they are a replacement of original calcite cements that are precipitated through a mimetic dolomitization process (Fig 12C, 12J, 12K and 12L) [71].

#### Mesostructure and microstructure interpretation

The co-occurrence of the traces of aquatic insects (caddisfly) and microorganisms in hand samples and thin sections reveals that the Shinekhudag Formation bioherms are predominantly controlled by the interaction of two distinct biologic communities. We interpret the main depositional controls of these bioherm forming components (caddisfly cases and microbialites) based on the depositional microfacies description of the packstone and boundstone samples.

The development of each caddisfly case is primarily controlled by the behavior of an individual larva/pupa [8]. The cases were built by the larvae/pupae to protect themselves from predators and to enhance their respiration efficiency, similar to their modern analogues [72]. Analysis of hundreds of caddisfly cases reveals that the Cretaceous Mongolian caddisflies constructed cylindrical tube-shaped cases during their larval stage using bits of debris from the surrounding environment [73,74]. Although the construction materials of the caddisfly cases are dominated by plant fragments (e.g., woody barks, branches, and leaves), they also incorporated other carbonate grains as well as clastic grains (e.g., ostracod valves, carbonate rock fragments, biotite, and feldspar) resulting in the complex architecture of cases (Fig 9). Therefore, the particles incorporated in the caddisfly case construction are a function of availability of materials in the surrounding lacustrine environment, thus they are a direct indication of paleoenvironment composition.

The two types of caddisfly case morphologies identified in hand samples and thin sections likely belonged to the same family of caddisfly since they all have prominent tubular shape and similar case architecture (Figs 7,9 and 11) [8]. However, the progressively increased size trends of these caddisfly cases (Fig 8) could reflect the size of the larvae/pupae that built and occupied them [73]. Thus, we argue the continuum of increasing sizes of the caddisfly cases represent growth stages of the caddisfly larvae [75]. Most modern caddisflies undergo four distinct case-building larval stages prior to their pupal stage and emerging as an adult insect [72].

Microfacies of the microlaminated micrite component reveal their primary depositional processes and controls. Laminated fabrics in carbonates are ambiguous in terms of the dominating controls on their sedimentation [21,22,76,77]. However, there are accretions of wavy, crinkled to semi-crinkled laminations, which form various shaped fabrics including spherical oncoids, finger-like columns, and cones with high inclination angles (Fig 12). Additionally, they trapped and bound flocculated micrite grains on their inclined surfaces, suggesting an adhesive chemistry [77,78]. A combination of these characteristics is highly indicative of a biotic fabric, and thus we conclude these microlaminates of the Shinekhudag Formation are primarily controlled by biologic activities rather than physical controls [22,66,79]. We interpret these microlaminated fabrics as microbialites or stromatolites, which were predominantly accreted by the interaction of microorganisms, perhaps filamentous cyanobacteria and/or algal mats [21,65,77,79,80].

Diagenetic microfacies of the packstones and boundstones reveal alteration processes associated with lithification of the bioherm [64]. The main diagenetic features of the packstones include primary interparticle and intraparticle pore spaces and their associated cements (Figs 10A, 10B, 10E and 11F). By contrast, the main diagenetic features of the boundstones include primary shelter and secondary vugular porosities and their associated cements (Fig 12B, 12C, 12J, 12K, 12L and 12M).

We interpret both the inter- and intraparticle porosities of the packstones, as well as shelter porosities of the boundstones, as syndepositional, developed during the deposition of the bioherms [67,81,82]. The interparticle porosities are developed within the original pore spaces between caddisfly cases and other allochems of the packstones (Fig 11Band 11F). The intraparticle pore spaces are developed within the interiors of the empty caddisfly cases (Fig 11F). Alternatively, the shelter porosities observed within microbialites are potentially resulted from the decay of bacteria (Fig 12B, 12C, 12J and 12K).

These primary pore spaces are filled with various types and stages of cements. Both the inter- and intraparticle porosities are completely filled with a single stage of fine to coarse grained, blocky calcite spars (Figs 10A, 10E and 11F). Additionally, the primary shelter porosities are filled with generations of cements including isopachous and fibrous calcite rims, evaporate gypsum, as well as granular microspar cements (Fig 12J and 12K). We interpret these cements to be precipitated immediately after deposition of the preceding growth of the microbialites. Evidently, all these primary pore space filling cements are syndepositional to early diagenetic as they are filling the primary pore spaces. Thus, we argue that these cements are potentially precipitated from subsequent carbonate-rich lake waters above the sediments that buried the bioherms [83,84]. The precipitation of different types of cements with respect to their chemical compositions (carbonate versus evaporitic gypsum cement) is possibly a result of the changes in the environment, particularly lake water chemistry [85–87].

Arguably, the secondary vugular porosities within the thin sections are developed during late diagenesis, a result from the changes in the environment enhancing dissolution processes (Fig 12B, 12L and 12M) [67,88]. The associated isopachous calcite and blocky calcite cements are potentially a result of meteoric diagenesis considering the thin sections are lack of burial diagenetic fabrics [63–66].

The mineral composition of the packstone and boundstones reveal bioherms are primarily composed of both dolomite (~60%) and calcite (~40%). The dolomite cements are replacement minerals based on the abundant calcite textures and the absence of primary dolomite texture in thin sections [69–71]. The original calcite cements were likely replaced by dolomite during an early diagenetic stage, as they are only found within the early diagenetic cements (Fig 12M) [64–66].

## Discussion

### Part 1: Evolution of the Tsagaansuvarga Basin (Late Jurassic through Early Cretaceous)

Widespread extension defined the main tectonic regime of eastern Mongolia and China during late Mesozoic time, expressed by nonmarine rift basins, bimodal volcanics, and associated metamorphic core complexes across the region [27,36,89]. These extensional basins are well preserved in the subsurface and partially exposed where they have been inverted by younger faulting events [29]. Rifting in southeast Mongolia was active from at least 155–126 Ma based on ^40^Ar/^39^Ar dating of ash beds in synrift fill [27,90].

Although late Mesozoic rifting is well known in eastern Mongolia and China, the western continuation of the extensional province is more enigmatic, particularly in northwest China where Jurassic-Cretaceous contraction is documented [25,91–93]. Late Mesozoic extension is now documented in the Gobi-Altai of western Mongolia, including the study area in the Tsagaansuvarga Basin (Fig 1) [25,37]. Extension in this area is largely coeval with the East Gobi Basin based on closely related structural style, correlative ages of the basin fill (126–155 Ma), and similar stratigraphic relationships (Fig 3) [27,30,90].

Large lake systems are recorded in thick (>2 km) synrift basin fill across the region; these deposits include oil shale intervals geochemically linked to produced oil, as well as synrift reservoirs largely in basin-margin clastic facies [43,94,95]. Broadly, synrift basin fill of the Tsagaansuvarga Basin gradually transitions from the lower interval, characterized by fluctuating-profundal facies association, to the upper interval, characterized by fluvial-lacustrine facies associations (Figs 4 and 5).

The lower stratigraphic interval of the Shinekhudag Formation in the Tsagaansuvarga Basin contains distal mudstone and oil shale beds interbedded with thin carbonates, turbidite sandstones, poorly sorted fan delta deposits, as well as littoral-zone bioherms, together displaying a thick aggradational stacking pattern. Such heterogeneous lithologies, deposited in a distal to littoral lacustrine environment, suggest the lake was deep [44,49,96]. The proximal alluvial fan deltas, interbedded with distal lake deposits, indicate steeply dipping topography, potentially along a basin-bounding normal fault [57,97,98]. A normal fault outcrops along the eastern margin of the basin and is juxtaposed with the offshore lake deposits, although its kinematics and relationship to the lake basin have not been fully investigated (Fig 2). Based on these lines of evidence, we interpret the lower interval was deposited in balance-filled, deep lake basin.

The overlying upper interval is distinguished from the underlying interval by an increase in bioturbation, a decrease in calcareous material, and higher energy sedimentary structures (Figs 4B,5E and 5F). This interval is also characterized by mudstone, siltstone, sandstone, and conglomerate beds. However, facies associations of this interval display a well-expressed progradational stacking pattern, indicating an increase in the sediment supply to accommodation ratio (Fig 4B) [44,45]. We therefore interpret the upper interval as deposited in an overfilled lake basin with open hydrology, which resulted in increased clastic grain size of the basin fill.

A change in depositional environments between the lower and upper intervals reveals the evolution of the lake basin type. Over time, the basin responded to the changing balance of main sedimentation controlling factors: potential accommodation space and sediment and water input [44,45]. The high potential accommodation and high sediment/water input during deposition of the lower interval resulted in a regional shoreline transgression and a balance-filled lake basin. This stage may coincide with a peak-rift time of when subsidence rate was high and basin accommodation was increasing [30]. Concurrently, the humid, subtropical climate was supplying sufficient sediment and water into the catchment area to balance the increased accommodation space [30,99]. In contrast, the lower potential accommodation and unchanged high sediment/water input during the deposition of the upper interval resulted in basinward-progradation of the shoreline, or an overfilled lake basin. This stage may coincide with an early rift-sag phase, when subsidence of the catchment area was decreasing and eventually overtaken by the continued high sediment and water input.

### Part 2: Stratigraphic context of bioherms

#### Development model of bioherms

We argue that the bioherms of the Lower Cretaceous Shinekhudag Formation developed as a result of symbiosis of two main biological communities: caddisfly larvae/pupae and microbialites. Together they played principal roles in the deposition of these bioherms similar to well-studied lacustrine bioherm examples in the western US, particularly the Eocene Green River Formation [13,14,19,20]. Therefore, we suggest naming them “microbial-caddisfly bioherms”. Both biogenic factors are equally important for understanding the development of microbial-caddisfly bioherms and both are controlled by ecological and environmental factors.

**Fig 13:**
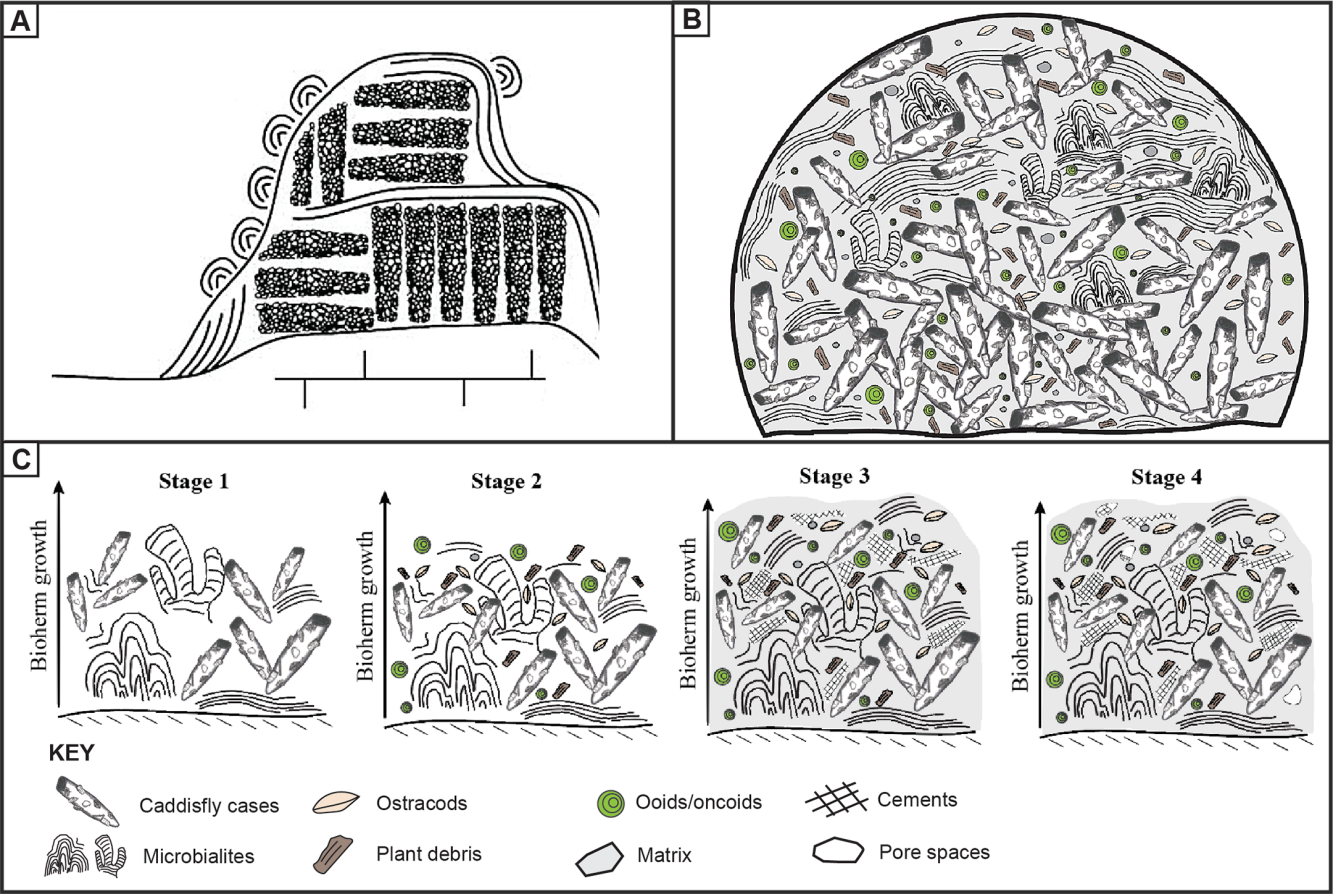
Development model of the microbial-caddisfly bioherms. (A) Development model of the Eocene Green River Formation (modified after Leggitt et al., 2001). (B) Development model of the Cretaceous Shinekhudag Formation. (C) Sequences of events that have resulted microbial-caddisfly bioherms of the Shinekhudag Formation.

Prior to this study, a development model of similar microbial-caddisfly bioherms was constructed, mainly focusing on the Eocene Green River Formation in North America [15,100]. The bioherms of the Green River Formation are composed of repeated rhythmic alternations of the well-arranged and uniformly-sized caddisfly cases and mm-scale microbial laminations [13,14,20]. Leggitt and others (2001) [19] rooted this association fundamentally to the biological behaviors of the caddisfly larvae/pupae and microorganisms. Modern caddisfly pupae communities often become coordinated and arrange their cases only during the annual pupation event [8]. Based on modern behavior, Leggitt and others argued that Eocene caddisflies similarly arranged their uniform-sized cases during yearly pupation events. After each pupation event, benthic microbialites grew on the empty case aggregates, slowly increasing the overall height of the bioherm until the next caddisfly pupation event. A combination of these repeated actions have resulted in the complex microbial-caddisfly couplet bioherms found in the Green River Formation (Fig 13A) [13,19].

Despite their similarities, there are notable differences in the Mongolian examples. In the Cretaceous Shinekhudag Formation, caddisfly cases are sub-aligned to unaligned, reflecting poorly organized or unorganized biotic action during bioherms development. The progressively increased size trends of these caddisfly cases reflect different growth (instar) stages of caddisfly larvae. Based on the biology of caddisfly larvae/pupae, the aggregations of non-uniformly sized empty cases reveal case-abandonment behavior during larval/instar stage [101]. Non-cyclic alternations of Mongolian caddisfly cases and associated microbialites are notably different than the Green River Formation couplets, as seen in thin section (Fig 12B). Instead, Shinekhudag Formation bioherms are typically preserved as aggregations of microbial laminae-bound caddisfly cases, without systematic cyclic alternations (Fig 13B). Due to these contrasting characteristics, the development mechanisms of the Shinekhudag Formation bioherms require a different interpretation than in the Green River Formation.

Based on the paragenetic relationship of the main bioherm building components including the grains, matrices, and generations of cements, we suggest the following sequences of events have resulted for bioherm development in the Early Cretaceous of Mongolia. First, simultaneous accumulation of abandoned caddisfly case aggregations and various growth forms of microbialites formed irregular frameworks with high baffling capacity (Fig 13C: Stage 1). Alternatively, it is possible that the decaying caddisfly cases provided stable growth sites hospitable for the microbialites (Fig 12B and 12H). The silken threads of the caddisfly cases are rich in protein, which provided nutrients to benthic microorganisms. According to Stewart and others (2010) [102], the caddisfly silk is made out of a protein named fibroin that contains high amount of carbon and phosphorus. The paleoenvironment that these framework-building organisms inhabited was rich in various types of detrital grains, resulting in complex architecture of the caddisfly cases. Second, these caddisfly/microbialite frameworks trapped various types of skeletal and non-skeletal intraclasts including plant fragments, ooids, ostracod valves, and rare clastic grains. These intraclasts are preserved as allochems in thin sections (Figs 10 and 13C: Stage 2). Third, the open pore spaces between the caddisfly case/microbialite frameworks and trapped grains were packed with micrite dominated matrices (Figs 10E, 10F, 11H and 13C: Stage 3). Concurrently, the remaining pore spaces were filled with the various types of syndepositional to early diagenetic cements in a short span of time that are precipitated from the carbonate rich lake waters (Figs 10,12 and 13C: Stage 3). At last, dissolution processes occurred in the bioherm due to changes in the environment. Secondary vugular porosities have developed during this event, and are filled by late diagenetic cements during meteoric diagenesis (Figs 12B, 12L, 12M and 13C: Stage 4) [66,67].

#### Paleoenvironment reconstruction

To reconstruct paleoenvironments, we focused on three main concepts: depositional setting (carbonate factory), lake water chemistry, and the lake basin dynamics. Depositional setting interpretations are largely based on fossil caddisfly case architecture analysis and overall bioherm composition. Lake chemistry interpretation depends on the composition of the main bioherm components: matrices and cements. Based on the stratigraphic expression of the basin fill, we interpret changes in lake dynamics of the Tsagaansuvarga Basin over time.

- Depositional setting

Caddisfly larvae and pupae capture a snapshot of their habitat by incorporating into their cases the most abundant materials available in their surrounding environment. Their cases reflect the composition of their habitat. Robust geological and paleontological analyses from the Eocene Green River Formation and other younger examples have successfully used caddisfly case architecture analysis for the paleoenvironment reconstructions (e.g., [13,14,19]). The microbial-caddisfly bioherms of the Shinekhudag Formation are some of the oldest known examples of fossil caddisfly cases (Early Cretaceous age) and record involvement of the caddisfly larvae/pupae, in contrast to younger examples. Notably, these caddisflies are the first documented to have used plant fragments as case building material.

The following bioherm characteristics elucidate complexities of their developmental setting. First, the coevolution of caddisfly cases and microbialites reveal a nutrient-rich, well-oxygenated environment, perhaps the littoral/photic zone of the lake with sufficient light for photosynthesis by the benthic microbial communities (Figs 11 and 12) [22,23]. Second, the abundant oolitic intraclasts and perfect spherical oncoidal fabrics suggest a shallow dynamic lake setting, above wave base (Fig 12G and 12I) [103]. Third, the caddisfly larvae/pupae introduced a large volume of detrital particles during bioherm development by building their cases with woody plant fragments (Fig 11A, 11B, 11D, 11G and 11H). These plant dominated case construction materials reveal the immediate composition of the paleoenvironment; they are likely derived from in-situ trees growing along the lake paleoshoreline [104].

**Fig 14:**
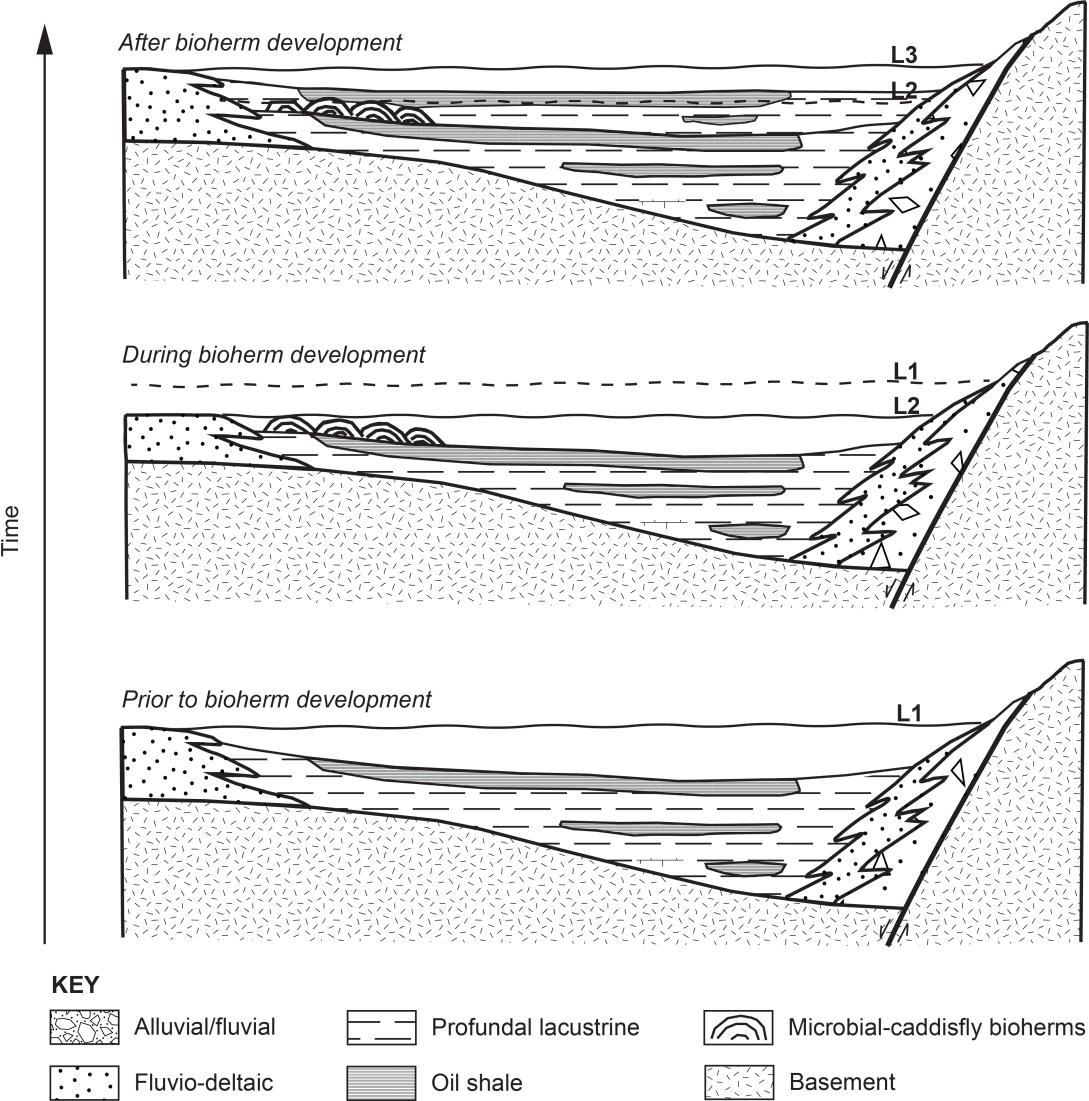
Schematic cross section of the lake dynamics and associated facies (adapted from Bohacs and Carroll, 2000).

Based on these key features, we interpret the microbial-caddisfly bioherms as the lacustrine carbonate shoreline facies, deposited potentially in a carbonate-ramp setting with variable littoral energy [18,50]. The subtle geometry of the ramp setting provided aggregation sites for caddisfly cases to build a framework (Fig 14). The bioherms may have linear arrangement in lake as they’re seen on the outcrop. Overall, our interpretation of Shinekhudag Formation bioherms agrees with key interpretations of the Green River Formation bioherms [13,14,16,19,100,105]. Their similar framework-supported, mound-shaped carbonate facies with closely associated biological components (caddisfly larvae/pupae and microbial communities) strongly suggest that they were developed in analogous carbonate factories [18,106].

- Lake chemistry

The carbonate dominated matrices and syndepositional to early diagenetic cements (e.g., isopachous & fibrous calcite cements) of the bioherms reveal details of the lake chemistry (Figs 10,11 and 12). The fine micrite matrices and calcitic cements precipitated in alkaline water rich in Ca/Mg and bicarbonate-ions (Figs 10,11 and 12) [107,108]. Additionally, a photosynthetic uptake of CO_2_ and/or bicarbonate of the filamentous cyanobacteria and algal mats could have increased the lake alkalinity locally, thus enhancing the precipitation of carbonates [21,22]. Abundant Ca/Mg ions are derived by erosion of the carbonate mineral-bearing basement, via surface water input from the surrounding hinterland, or Ca-rich spring waters [49,109]. However, we have not observed large faults near the microbial-caddisfly bioherm outcrops or tufa fabrics that indicate local spring water discharge.

The lake water also might have contained sulfates, based on microscopic-scale gypsum crystals found from the bioherm pore spaces (Fig 12J and 12K). In general, evaporitic minerals in the lacustrine systems are indicative of saline-hypersaline lake water, often a result of closed basin hydrology [44,45,86]. In these cases, the saline-hypersaline lake conditions may explain dolomite-dominated composition of the matrices and early diagenetic cements of the bioherms. This idea of early dolomitization is debatable, due to the limited extent of evaporitic minerals in the Tsagaansuvarga Basin as well as the general controversy regarding dolomite formation [108,110]. However, it could explain our results, and it requires further attention. In summary, we interpret that the paleo-lake chemistry was saline to hypersaline, rich Ca/Mg as well as biocarbonate ions, and relatively alkaline during the microbial-caddisfly bioherm deposition.

- Lake dynamics

A close look at the microbial-caddisfly bioherms and the surrounding facies of the Shinekhudag Formation reveals a dynamic lake setting (Fig 4B and 4C). The microbial-caddisfly bioherms are found within the lower interval (1800–2862 m) of the Shinekhudag Formation. The microbial-caddisfly bioherms are encased in a thick and discrete interval of oil-shale, interpreted as a classic distal lake deposit (Figs 4C,5A,5 B, and 6) [46,111,112]. We interpret the lower interval as deposited in a balance-filled basin type, during a regional high-stand of the lake (Fig 14). In contrast, our interpretations of depositional model as well as the paleoenvironment reconstruction both conclude the microbial-caddisfly bioherms are a littoral, nearshore lacustrine facies, deposited along the paleo lake shoreline [18,50].

This stratigraphic juxtaposition is distinct compared to analogous settings, especially because the linear biohermal belt (~1 km along strike) occurred without disrupting the oil-shale accumulation (Figs 2,6A, 6B and 6C). This oil-shale interval suggests that the stratified, anoxic bottom was not disturbed during the bioherm development (Fig 14). Thus, we interpret that the microbial-caddisfly bioherms were deposited during a brief, rapid regression of a shrinking lake. Materials incorporated in the caddisfly case construction support this interpretation by revealing low pulses of clastic grains with rare biotites and feldspars that are likely derived from syndepositional volcanism (Fig 11E and 11G). This building material selection may not be coincidental, especially if the lake hydrology was closed. Compared to the lacustrine microbialite facies-model of the Eocene Green River Formation, this theory of the hypersaline lake condition is controversial [15], but is a viable scenario that could explain results presented in this study.

In summary, the interpretations presented above suggest rapidly changing lake basin dynamics, transitioning from a balance-filled to an underfilled lake basin type and back, in a short span of time (Fig 14). This rapidly changing lake basin type could either be controlled by superimposed paleoclimatic fluctuation (increased evaporation), and/or tectonically driven rapid basin subsidence, resulting in increased accommodation [44].

### Part 3: Caddisfly taxonomic classification

Detailed investigation of the fossil caddisfly cases of the microbial-caddisfly bioherms of the Shinekhudag Formation reveals underwater behavior of the Cretaceous caddisfly larvae. In 1968, Russian entomologist Sukatsheva reported the first discovery of fossil caddisflies and their cases from the Berriasian and Aptian of Mongolia [9,113,114]. However, no fossil caddisflies or their cases were reported from the Shinekhudag Formation or the Tsagaansuvarga Basin prior to our study. Here, we propose family level taxonomic identification of a caddisfly based on case architecture, size, and geometry without the associated fossil larvae.

Generally, the caddisflies are classified as *Insecta Trichoptera* and further divided into three main suborders: cocoon-making caddisflies (*Spicipalpiai)*, retreat-making caddisflies (*Annulipalpia*), and portable case-makers (*Integripalpia*). Each of these suborders’ cases can be distinguished by their definitive structure [115].

We use the following six main characteristics of the Shinekhudag Formation fossil cases to help tentatively assign their maker’s family. First, the caddisfly communities of the Shinekhudag Formation lived around the littoral zone of a lake (Fig 14). Second, both types of caddisfly cases (e.g., straight and curved) have similar tube shapes that are perfect circles in a cross section (Figs 9 and 11). Third, their lengths range from 7 to 21 mm and their width ranges between 1.5 to 2.5 mm (Fig 8). Fourth, the poorly aligned to unaligned case groups reveal caddisfly larvae/pupae non-stationary life mode (Fig 13). Finally, progressively increasing case size trends (Fig 8) reveal a case-abandonment behavior [101]. In modern examples, the caddisfly larvae/pupae abandon their cases under number of conditions, including an environmental stress, a predator threat, or a failure of cases to meet their convenience [75,116].

**Fig 15:**
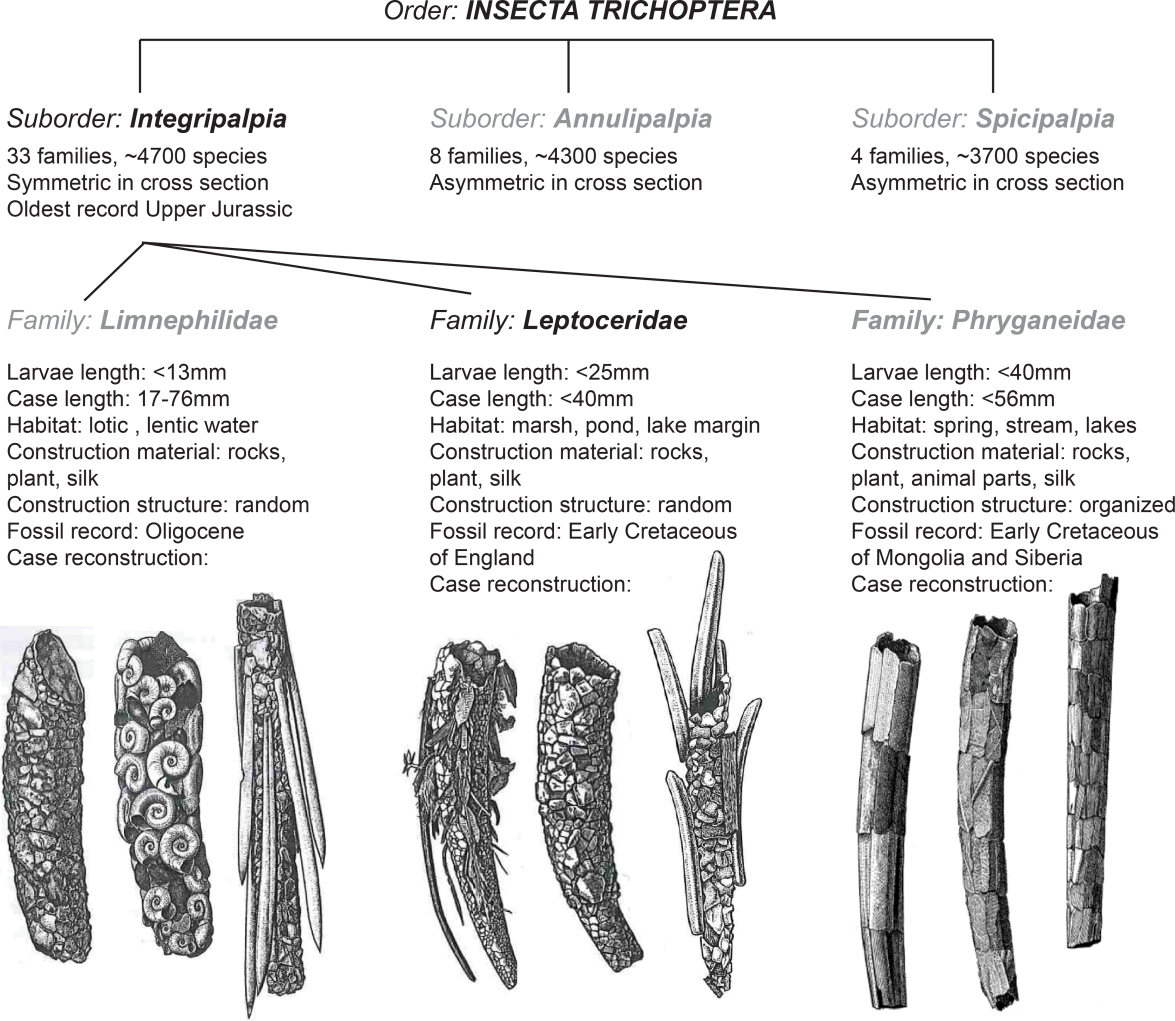
Caddisfly suborders and families (modified after Wiggins, 2004; Holzenthal et al., 2007).

Both the first and third characteristics (symmetrical, tubular-shaped cases and non-stationary life mode) are enough to assign the cases to the suborder *Integripalpia*—the portable case makers (Fig 15) [19,117]. In contrast, protective cases of the other two suborders of caddisflies (*Spicipalpiai* and *Annulipalpia*) have asymmetric, oval-shapes in cross section resulting from their stationary life-mode [8]. The suborder *Integripalpia* includes total 33 families, but only three (*Leptoceridae*, *Limnephilidae*, and *Phryganeidae)* are adapted to the littoral lake zone, and build tube shaped portable cases with detrital plant and rock fragments (Fig 15) [8,73].

The earliest insect fossils and cases of the *Leptoceridae* and *Phryganeidae* are dated to Early Cretaceous time, but the *Limnephilidae* appears in the fossil record much later, from the Oligocene (Fig 15) [72,114]. Thus, we focus on the *Leptoceridae* and *Phryganeidae* families for the tentative assignment. Unfortunately, distinguishing between these two families based on their case morphology is challenging, and interestingly, these two families both occasionally show case-abandonment behavior during their larval/pupal stages [75,101]. The only difference between these two families is the detailed architecture of their case construction. The modern family of the *Phryganeidae* builds neatly constructed, organized cases by using approximately even-sized, homogeneous types of materials. In contrast, the *Leptoceridae* build randomly constructed cases by using various-sized, heterogeneous types of materials [8,75,118].

Overall, based on our observation, the caddisfly cases of the Shinekhudag Formation are composed of plainly constructed cases with various-sized, heterogeneous fragments by using the most immediate materials from the surrounding environment (*Fig 9)*. Therefore, we conclude that the caddisfly cases of the Shinekhudag Formation are probably built by the family of *Leptoceridae*, analogous to Lower Cretaceous caddisfly cases from South Korea [10].

## Conclusion

The first detailed description of microbial-caddisfly bioherms of the Shinekhudag Formation reveals important aspects about lacustrine paleoenvironments during the late Mesozoic of southwestern Mongolia. A combination of the depositional environment interpretation of the basin fill, the development model of microbial-caddisfly bioherms, and the caddisfly case architecture analysis indicates a period of unstable lake condition and a highly dynamic basin setting. Additionally, architectural analysis of the caddisfly cases contributes to an ancestral knowledge of caddisfly underwater behavior and demonstrates that caddisflies are important environmental indicators.

The Lower Cretaceous microbial-caddisfly bioherms of the Shinekhudag Formation are one of the earliest examples documented globally. These bioherms record caddisfly case-microbialite dominated frameworks and their baffled/trapped grains, as well as matrices and cements that packed/cemented them together. The main bioherm building components (i.e., microbialites, caddisfly cases, cements, and matrices) suggest relatively alkaline, saline lake water that was rich in Ca/Mg and bicarbonate ions. It is also possible the lake water salinity was increased periodically to a hypersaline condition based on the early dolomitization process as well as the presence of minor gypsum cements that are inferred from the thin sections. The linearly arranged (~1 km) biohermal belt was developed along the lake paleolake shoreline, within a littoral zone. Based on the stratigraphic relationship of the bioherms as well as the encasing oil-shale intervals, we argue the microbial-caddisfly bioherms were developed in a rapidly shrinking lake, a result of the lost balance of accommodation space and sediment plus water supply.

Additionally, the caddisfly case architecture analysis reveals the interesting facts about lacustrine paleoenvironments. The fossil cases of caddisflies belong to a single species of the caddisfly larvae based on their case morphology. The plant materials, particularly wood pieces found from the caddisfly cases, represent the oldest record of fossil plant fragment for caddisflies, and are derived from lake shoreline vegetation. Also, a few biotite and feldspar fragments that are found from their cases suggest syndepositional magmatism. Arguably, the construction material selection of caddisfly larvae/pupae is mainly a factor of an availability of the detrital grains from their surrounding environment. These materials support our interpretation of a lake basin with closed hydrology during the bioherm development. Without the associated fossil larvae/pupae, we tentatively attribute these fossil cases to the caddisfly family of *Leptoceradae*, a species that are previously known from Early Cretaceous lacustrine systems of South Korea [10].
